# The Impact of Voluntary Exercise on Stroke Recovery

**DOI:** 10.3389/fnins.2021.695138

**Published:** 2021-07-12

**Authors:** Klara J. Lohkamp, Amanda J. Kiliaan, Justin Shenk, Vivienne Verweij, Maximilian Wiesmann

**Affiliations:** Department of Medical Imaging, Anatomy, Radboud University Medical Center, Preclinical Imaging Center – PRIME, Radboud Alzheimer Center, Donders Institute for Brain, Cognition and Behaviour, Nijmegen, Netherlands

**Keywords:** stroke, exercise, neuroimaging, polarized light imaging, mouse model

## Abstract

Stroke treatment is limited to time-critical thrombectomy and rehabilitation by physiotherapy. Studies report beneficial effects of exercise; however, a knowledge gap exists regarding underlying mechanisms that benefit recovery of brain networks and cognition. This study aims to unravel therapeutic effects of voluntary exercise in stroke-induced mice to develop better personalized treatments. Male C57Bl6/JOlaHsd mice were subjected to transient middle cerebral artery occlusion. After surgery, the animals were divided in a voluntary exercise group with access to running wheels (RW), and a control group without running wheels (NRW). During 6 days post-stroke, activity/walking patterns were measured 24/7 in digital ventilated cages. Day 7 post-surgery, animals underwent MRI scanning (11.7T) to investigate functional connectivity (rsfMRI) and white matter (WM) integrity (DTI). Additionally, *postmortem* polarized light imaging (PLI) was performed to quantify WM fiber density and orientation. After MRI the animals were sacrificed and neuroinflammation and cerebral vascularisation studied. Voluntary exercise promoted myelin density recovery corresponding to higher fractional anisotropy. The deteriorating impact of stroke on WM dispersion was detected only in NRW mice. Moreover, rsfMRI revealed increased functional connectivity, cerebral blood flow and vascular quality leading to improved motor skills in the RW group. Furthermore, voluntary exercise showed immunomodulatory properties post-stroke. This study not only helped determining the therapeutic value of voluntary exercise, but also provided understanding of pathological mechanisms involved in stroke.

## Introduction

Stroke is the fifth leading cause of death and the leading cause of long-term disability in the US in 2020 (Ahmad and Anderson, 2021; Jaillard, 2021). Ischemic stroke accounts for the majority of stroke cases (80%) and may cause severe ischemic brain damage (Donkor, 2018). Management of ischemic stroke focuses primarily on time critical thrombolysis, recanalization therapy and treatment of symptoms (Sehatzadeh, 2015; Hankey, 2017). Efficacy of available therapies is insufficient considering the high number of stroke survivors suffering from persistent symptoms, like disability (40%) (Hankey, 2017). Beside antithrombotic agents, drugs based on other mechanisms, like neuroprotectants, failed clinical trials (Chen and Wang, 2016). Alternatives to pharmacotherapeutic treatments are therefore urgently needed (Chen and Wang, 2016).

The main cause of stroke-related neurological deficits is focal tissue damage. Due to severe lack of oxygen and glucose supply by proper blood circulation, the ischemic core of the stroke is an irreparable area. Subsequently, a cascade of deteriorating mechanisms in the stroke territory is initiated, such as acute neuroinflammation (e.g., IBA-1+ microglia/macrophages), apoptosis and edema. The restoration of blood flow and vascular integrity (e.g., measured by glucose transporter-1 positive vessels (GLUT-1+)) can rescue damaged, but salvageable neurons in the penumbra, which is surrounding the apoptotic core of the stroke territory. White matter (WM) is very susceptible to ischemia, since it receives little collateral blood supply (Wang et al., 2016). In the 1st week after stroke WM and grey matter (GM) continues to degenerate after stroke, resulting in persistent damage of functional brain network connectivity (Guggisberg et al., 2019). More stroke studies in search of neurobiological mechanisms that might facilitate recovery of neuroimaging biomarkers are needed. In addition, the restoration of cerebral blood flow (CBF) can rescue damaged neurons in the penumbra (Astrup et al., 1981). Equally important is to inhibit neuroinflammation, improve neuro- and synaptogenesis, and vascular health.

Exercise has long been recognized to be effective in prevention of several cardiovascular diseases (CVD), including stroke (Orkaby and Forman, 2018). In addition, physical exercise is counteracting the most common (vascular) risk factors of stroke, including hypertension, atherosclerosis, and obesity (Orkaby and Forman, 2018). In clinical studies multiple positive effects of exercise have been found in stroke patients, including improved arm function, balance and gait (Alves et al., 2016; Boehme et al., 2017). Another fMRI study showed that exercise intervention for chronic stroke survivors improved mobility and cognition of the participants (Landsmann et al., 2016). Long-term, personalized exercise programs are however not yet implemented in clinical practice, whereas stroke patients may benefit from exercise induced neurologic rehabilitation. Several neuroprotective mechanisms are associated with improved stroke outcome. In particular, elevated vascular endothelial growth factors (VEGF) as well as brain-derived neurotrophic factor (BDNF) positively influenced neuronal survival and angiogenesis (Kramer et al., 2019). Additionally, there are indications that exercise attenuates detrimental effects by activated microglia and macrophages acute after stroke induction (Lovatel et al., 2014).

A persistent lack of knowledge exists concerning efficacy of voluntary exercise in recovering of structural and functional brain networks and subsequent functional recovery after stroke (Wu et al., 2017). The cerebral capability for functional changes, such as better connectivity between brain regions, as well as structural changes, including better WM integrity, has been reported to be crucial for motor function recovery (Wu et al., 2017). Though progress has been made in studying the impact of physical exercise on stroke rehabilitation, no studies are available investigating whether exercise can help to attenuate stroke induced brain network damage, impaired WM integrity and related motor deficits.

Therefore, the current study sought to determine the efficacy of voluntary exercise to attenuate brain damage in the acute phase after stroke. We intentionally chose this early time point, since several deteriorating effect of stroke, e.g., on edema volume, cerebral blood flow, and cerebral microstructure often diminish within the 1st days in stroke induced mice, independent of treatments (Barca et al., 2021). Since these parameters are belonging to our primary outcome measures, we decided to perform the experiments within 1 week after surgery. We hypothesize, that voluntary exercise is positively influencing (molecular) mechanisms crucial for stroke recovery, taking into account CBF, structural and functional (f)MRI parameters, molecular markers (VEGF, BDNF), vascular density/quality (GLUT-1+ vessels and intensity), neuroinflammation (IBA-1+ microglia and macrophages) and behavioral read-outs in a mouse stroke model 1 week post-stroke. Notably, in addition to diffusion tensor imaging (DTI), as an *in vivo* read-out for WM integrity, we performed for the first time the innovative polarized light imaging (PLI) in murine *postmortem* brains (Axer et al., 2011; Mollink et al., 2017). By using PLI we can investigate the amount and orientation of myelin with much higher spatial resolution than equivalent estimates derived from *in vivo* DTI, revealing insight into anatomical changes. This study not only investigates the therapeutic value of voluntary exercise after stroke, but it also foresees in better understanding of stroke pathology and rehabilitation.

## Materials and Methods

### Animals

**FIGURE 1:**
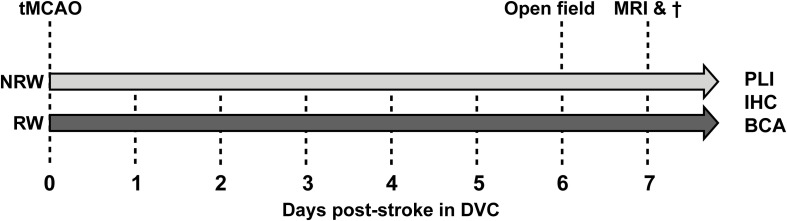
After right transient middle cerebral artery occlusion (tMCAO), mice were randomly allocated into a running wheel (RW) group and a no running wheel (NRW) group. RW mice had 24/7 access to voluntary exercise, while control mice had no running wheel in the cage. Activity/walking patterns of each mouse was individually monitored in digital ventilated cages (DVC) 24/7 for 1 week. Moreover, an open field test was performed to further investigate locomotion and activity. Immediately after MRI scanning, animals were sacrificed and brains were obtained to perform post-mortem immunohistochemistry (IHC), biochemical analysis (BCA), and polarized light imaging (PLI).

The present study has been performed in the Preclinical Imaging Center (PRIME) of the Radboud university medical center (Radboudumc, Nijmegen, Netherlands). If possible, experiments were performed double-blinded using 3 months old C57Bl6/JOlaHsd mice (*n* = 15) (Harlan Laboratories Inc., Horst, Netherlands). Sample size was determined by power analysis before the start of the experiment (Supplementary material). After stroke induction, mice were single housed in Digital Ventilated Cages (DVC) (Tecniplast S.p.A., Buguggiate (VA), Italy) which contained corn based bedding material (Bio Services, Uden, Netherlands), wood wool nesting material (Bio Services, Uden, Netherlands), and a mouse igloo (Plexx, Elst, Netherlands). All mice had *ad libitum* access to standard pelleted food (Ssniff rm/hV1534-000, Bio Services, Uden, Netherlands) and autoclaved water. Anxiety-related behavior (open field) did not differ from group-housed stroke-induced mice in a previous study, indicating that single housing had no significant impact on behavior (Calahorra et al., 2019). The animals were housed in a room imitating an 12h light-dark cycle (lights on at 7:00 a.m.), having constant humidity (55 ± 10%) temperature (21 ± 1°C), and background music. The study was performed according to the ARRIVE (Kilkenny et al., 2010) and (updated) STRIVE guidelines (Stroke,, 1999; Fisher et al., 2009; Boltze et al., 2016). After stroke induction animals were randomly allocated into two groups: (1) an exercise group, including animals with a running wheel (RW) in their DVC (GYM500 activity wheel, Tecniplast S.p.A., Buguggiate (VA), Italy) and (2) a no running wheel (NRW) group, including animals without a running wheel in their DVC. The behavioral experiments (open field test) as well as the MRI imaging were performed during daytime between 7:00 a.m. and 18.00 p.m. Excluded mice per experment are shown in Supplementary Table 1. The study design is described in detail in Figure 1.

### Transient Middle Cerebral Artery Occlusion

As described elsewhere (Wiesmann et al., 2017) a right (ipsilateral) transient middle cerebral artery occlusion (tMCAO) (30 min) was induced in male C57Bl6/JOlaHsd mice. Animals were anesthetized with an induction dose of 2–3% isoflurane (Abbott Animal Health, Abbot Park, IL, United States) in a 2:1 air and oxygen mix. During surgery isoflurane was reduced to a maintenance dose of 1.5–2%. Body temperature was kept at constant levels by using a heating pad. Cerebral blood flow of the MCA region was monitored with a Laser Doppler Flow probe (moorVMS-LDF2, Moor Instruments), which was fixed on top of the skull. A decrease of CBF (>80%) during the tMCAO was indicative of successful stroke induction. Via a neck incision, a coated 7-0 monofilament (tip diameter 190 to 200 um, coating length 2 to 3 mm, 70SPRePK5, Doccol Corp., Sharon, MA, United States) was introduced into the right common carotid artery and pushed upwards along the right internal carotid artery, where it occluded the origin of the right MCA in the Circle of Willis (proximal MCA occlusion). After 30 min, the filament was withdrawn to allow reperfusion of the MCA region. One day after stroke induction, all animals received a subcutaneous Carprofen injection (Rimadyl, Pfizer Animal Health B.V., Capelle aan den IJssel, Netherlands) to prevent postoperative infection and pain. During the 1st week after stroke *N* = 5 mice died (33% mortality).

### Digital Ventilated Cages

After stroke induction all animals were single-housed in digital ventilated cages (Tecniplast S.p.A., Buguggiate (VA), Italy) which record mouse activity and mouse centroids 24/7 after stroke induction (Voikar and Gaburro, 2020). All system specific details were previously described (Iannello, 2019; Shenk et al., 2020). Day- and nighttime activity metrices were calculated, each time interval lasting for a 12h. The average distance walked was calculated as total distance covered by the mouse within day- and nighttime. Based on the centroids and x,y-coordinates distance, velocity, laterality, and occupation of running wheel was computed.

#### Mice Activity Metrics

As previously described a sensing board, equipped with 12 capacitive-based electrodes underneath each DVC, measured the activity and centroids of each individual mouse (Iannello, 2019). As soon as an animal is moving in the cage, the electrical capacitance of the proximity sensors are influenced by the dielectric properties of matter in close proximity to the electrode. Consequently, animals moving across the electrodes are detected and recorded as change in capacity over a limited time interval. An activity event describes the absolute value of the difference between two consecutive measurements for each electrode that is compared with a set threshold to control for noise. For the *x*-, *y*-value calculation, the mouse position is estimated in the average position between the centers of the active electrodes weighted by using change in capacity. For the final analysis of relative cage activity, the electrode under the running wheel (electrode 6) was excluded and we calculated the average activity of 11 electrodes per 12h day- and nighttime for each mouse, respectively. The heatmap in Figure 3A represents the average home cage activity per hour in RW and NRW mice recorded during 6 days post-surgery.

#### Distance Walked and Average Speed in Home-Cage

The mouse position on the cage floor is monitored based on the centroids, and the walked distance is calculated via a sum of the Euclidean distance of the mouse centroid subsequentially framed within the considered time interval (Iannello, 2019). The average speed is computed by dividing the distance walked by the duration the used time interval (Iannello, 2019).

#### Occupation of Running Wheel

The cage area can be used to analyze spatial preference of mice in the cage. The running wheel occupation metric measures the relative time a mouse spent in the running wheel area (via electrode 6 located under the running wheel), for further details see Iannello (2019). The running wheel is fully covering the sensor and the space between cage floor and running wheel is not accessible for the mice. Therefore, the recorded occupation was only derived by movements of the running wheel. The relative time mice spent on average per minute during day- and nighttime (12h interval) in the running wheel sensor area was calculated.

#### Turn Angle and Laterality

Turn angle and laterality was computed with the Traja Python package which has been earlier described in detail (Shenk et al., 2020). In short, we calculated the a laterality index (LI) by dividing the number to right turns (R) by the sum of right and left turns (L) ($LI=\frac{R}{R+L}$) (Shenk et al., 2020). Turn angle and laterality are computed based on walking patterns (*x*−, *y*−values in the cage), and is a measure of motor functions.

### Open Field

The open field task was performed to evaluate spontaneous behavior and exploratory locomotion as previously described (Wiesmann et al., 2017, 2018; Calahorra et al., 2019). Mice were placed in a squared arena (45 × 45 × 30 cm) and had the chance to freely explore it for 10 min. Animals were video-taped and a tracking software (EthoVision, XT 15, Noldus, Wageningen, Netherlands) automatically calculated different parameters (Streijger et al., 2005).

### Imaging Protocol: ASL, DTI, and rsfMRI

MRI measurements were performed 6 days after surgery with an 11.7T BioSpec Avance III small animal MR system (Bruker BioSpin, Ettlingen, Germany) operating on Paravision 6.0.1 software (Bruker, Karlsruhe, Germany). The animals were anaesthetized with 3.5% incubation dose of isoflurane and during the MRI scan the dose was kept at 1.8% in a 2:1 air and oxygen mix (Abbott Animal Health, Abbot Park, IL, United States). One mouse of the NRW group was excluded from the analysis, because the scan showed, e.g., movement-, EPI-related artifacts (Supplementary Table 1).

Cerebral blood flow (CBF) was assessed with a flow-sensitive alternating inversion recovery arterial (FAIR) technique as previously described (Calahorra et al., 2019). The regional CBF was calculated with the same protocol as described previously (Calahorra et al., 2019). Functional connectivity was measured with rsfMRI as described elsewhere (Calahorra et al., 2019).

Diffusivity measures of the brain were evaluated via DTI, measuring mean diffusivity (MD) and fractional anisotropy (FA) following a standard protocol (Calahorra et al., 2019). FA is a value, indicative for myelination and fiber density of WM and mean diffusivity MD is an inverse measure of membrane density (Le Bihan et al., 2001; Feldman et al., 2010; Alexander et al., 2011).

Stroke infarct size was calculated from the DWI images of the MRI scans using ImageJ (version 1.47, National Institute of Health, Bethesda, MD, United States). RW and NRW mice exhibited similar infarct size (*F*(1,8) = 0.27, *p* < 0.62) (Supplementary Figure 1). All MRI parameters were motion corrected. Detailed MRI imaging sequences are listed in Supplementary Table 2.

### (Immuno)histochemistry: Brain Tissue Preparation

After neuroimaging, the animals were sacrificed via transcardial perfusion with 0.1 M phosphate-buffer saline (PBS) followed by 4% paraformaldehyde in 0.1 M PBS (pH 7.4) at room temperature. Parts of the frontal brain of both stroke affected (ipsilateral) and control (contralateral) hemisphere, were snap-frozen and used for qPCR analysis. The remainder of the brains was postfixed overnight in 4% paraformaldehyde 4°C and transferred to 0.1 M PBS containing 0.01% sodium azide the next day. Approximately 24 h previous to cutting, the brains were put into 30% sucrose in 0.1 M phosphate buffer. The brains were cut with a microtome (Microm HC 440, Walldorf, Germany) having an object table for freezing-sectioning at −60°C. According to the atlas of Franklin and Paxinos (Paxinos and Franklin, 2004) eight series of 30 μm frontal sections between bregma −0.1 to −4.36 were cut and stored in 0.1 M PBS with 0.01 sodium azide for multiple immunohistochemical stainings and PLI. For each immunohistochemical procedure and PLI one mouse was excluded from analysis, because of damaged tissue sections due to large stroke lesions (Supplementary Table 1).

#### (Immuno)histochemistry: GLUT-1 and IBA-1

Immunohistochemical stainings were performed to assess (1) the amount of glucose transporter-1 (GLUT-1) as indicator for blood vessel quality and (2) activated microglia and macrophages as measure for neuroinflammation by ionized calcium-binding adapter molecule 1 (IBA-1). As described in detail previously, immunohistochemistry was performed using standard free-floating labeling procedures (Janssen et al., 2016). GLUT-1 was visualized using polyclonal rabbit anti-GLUT-1 antibody (1:40,000, Chemicon AB 1340, Chemicon International, Inc., Temecula, CA, United States) and as secondary antibody donkey anti-rabbit biotin (1:1500 Jackson ImmunoResearch, West Grove, PA, United States). For IBA-1 a primary antibody against IBA-1 polyclonal goat anti-IBA-1 (1:3000; Abcam, Cambridge, United Kingdom) was used. Donkey antigoat biotin (1:1500; Jackson ImmunoResearch) was used as a secondary antibody. Representative images of the ipsilateral and contralateral brain hemisphere were made with ImageJ (version 1.47, National Institute of Health, Bethesda, MD, United States) stitching plugin (Preibisch et al., 2009), using three images that were taken with 5× magnification of different ROI.

### Quantification: GLUT-1 and IBA-1

Two double-blinded researchers quantified the stainings (bregma: −1.46 to −2.30) according to the atlas of Paxinos and Franklin (2004). Representative images were taken on images with 5× magnification using Axio Imager A2 (Zeiss Germany). Several ROI were manually selected in (GLUT-1 + IBA-1: Cortex (bregma 0.62 & −1.94), hippocampus (−1.94), corpus callosum (0.62), basal ganglia (0.62), and thalamus (−1.94)) Image J (National Institute of Health, Bethesda, MD, United States). Particles (GLUT-1, IBA-1) were automatically detected and counted by Image J (particle number per area). Moreover, the stained area was calculated relative to the manually drawn ROI.

### Polarized Light Imaging

Polarized light imaging (PLI) was performed to assess fiber density and fiber spreading in several ROI (bregma 0.14; 0.50 and/or −1.94: anterior commissure, basal ganglia, cerebral peduncle, corpus callosum, cortex, external capsule, fimbria, fornix, hippocampus, internal capsule, motor cortex, optic tract, somatosensory cortex, thalamus) in the stroke affected (ipsilateral) and control (contralateral) hemisphere, respectively. The transmission of polarized light can be used to visualize and quantify the orientation and inclination angles at every pixel of the imaged specimen by utilizing the birefringent properties of the myelin sheaths aligned around the axons (Axer et al., 2011; Mollink et al., 2017). PLI was performed on 30 μm slices that were mounted on uncoated glass. After a drying period of 24 h at 37°C, the sections were cover-slipped with polyvinylpyrrolidone mounting medium. PLI was performed 1 week after mounting to allow them to dry. Images were taken on a Axio HV microscope (Zeiss, Germany) equipped with an RGB camera (AxioCam ERc 5s, Zeiss, Germany), a rotating polarizer, a quarter wave plate, a stationary polarizer and a white LED light source. First, light passes through a linear polarizer combined with the quarter wave plate which axis was orientated in 45° angle with respect to the polarizer plane. As such, polarized light is created, passing through the specimen followed by another polarizer, which captures the change in polarization induced by tissue birefringence. A raw image dataset comprised 9 sequential images at rotation angles between 0 and 160. Images for background correction were acquired in the same manner for every imaging session. Post-processing of the raw images was performed with Matlab R2018b. After background correction, different parameter maps were derived by fitting the PLI images to Jones Calculus, leading to in-plane orientation, fiber orientation, retardance, inclination, and transmittance map. Myelin density and orientation was quantified with ImageJ (version 1.47, National Institute of Health, Bethesda, MD, United States). ROI inside the retardance and dispersion maps were manually selected (bregma: 0.14; 0.50; and/or −1.94). In the graphs, for both retardance and dispersion the normalized mean gray values are represented using the mean gray value of the contralateral ROI of NRW mice as reference value.

### qPCR

Ipsilateral (stroke hemisphere) and contralateral (control hemisphere) parts of snap-frozen frontal brain tissue was used to analyze mRNA expression of postsynaptic density protein 95 (Psd-95), brain derived neurotrophic factor (Bdnf), and glucose transporter 1 (GLUT-1), and synaptophysin using qPCR as previously described (Calahorra et al., 2019). Glyceraldehyde-3-Phosphate Dehydrogenase (GAPDH) and beta-2 microglobulin (B2m) were used as normalizing genes. The sequences of primers are shown in Supplementary Table 3. Vascular endothelial growth factor (Vegf) levels tended to be higher in RW mice in comparison to NRW mice (Supplementary Figure 2: *F*(1,6) = 5.0, *p* < 0.068).

### Statistics

All data collected in this experiment were assessed via a randomized and double-blinded procedures. Statistical analysis was performed with SPSS 25 (IBM SPSS Statistics 25, IBM Corporation, Armonk, NY, United States).

All data were normally distributed. Multivariate and repeated measures analysis of variance (ANOVA) with Bonferroni correction for multiple testing (e.g., number of ROI) was conducted to examine group and time differences. To determine inter-hemispheric difference between the ipsilateral and contralateral hemisphere, the data were split in treatment groups before running repeated measures ANOVAs with Bonferroni correction. All data are represented as mean ± standard error of mean (SEM). #, 0.05 < *p* < 0.08 (tendency); ^∗^, *p* ≤ 0.05; ^∗∗^, *p* ≤ 0.01; ^∗∗∗^, *p* ≤ 0.001

## Results

The complete study design, including the behavioral testing, MRI imaging, and post-mortem analysis is displayed in Figure 1.

### Body Weight and Food Intake

**FIGURE 2:**
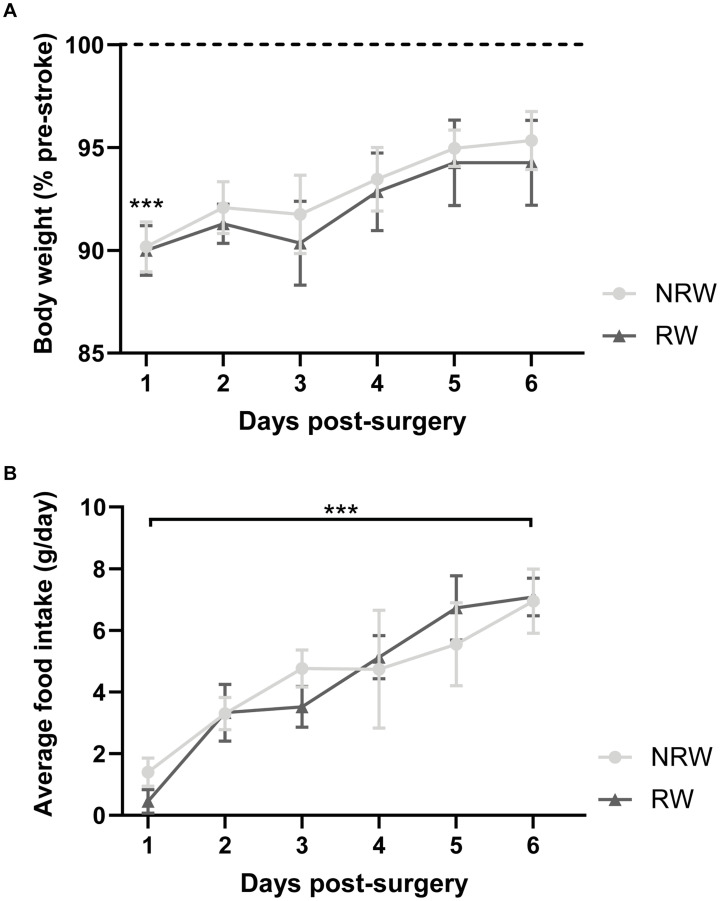
**(A)** Body weight relative to pre-stroke weight and **(B)** food intake per day in running wheel (RW) and no running wheel mice (NRW). RW: *N* = 5, NRW: *N* = 5; values represented in mean ± SEM. ***p ≤ 0.001.

Body weight dropped significantly 1 day post-stroke both in running wheel (RW) and no running wheel mice (NRW) (Figure 2A: *F*(1,8) = 14.2, *p* < 0.001). The average body weight pre-surgery was not significantly different between the groups (RW: 26.6g (±1.96) and NRW: 28.0g (±0.77); *F*(1,8) = 0.466, *p* < 0.514). At day 6 post-surgery, in all mice no difference between pre- and post-surgery body weights was detected (Figure 2A: *F*(1,8) = 14.2, *p* < 0.087). Body weight did not increase between 1 and 6 days post-surgery (Figure 2A: *F*(1,8) = 14.2, *p* < 0.077). Food intake significantly increased over 6 days after surgery in both groups (Figure 2B: *F*(5,40) = 5.6, *p* < 0.001). No group differences in body weight and food intake were detected.

### Activity

**FIGURE 3:**
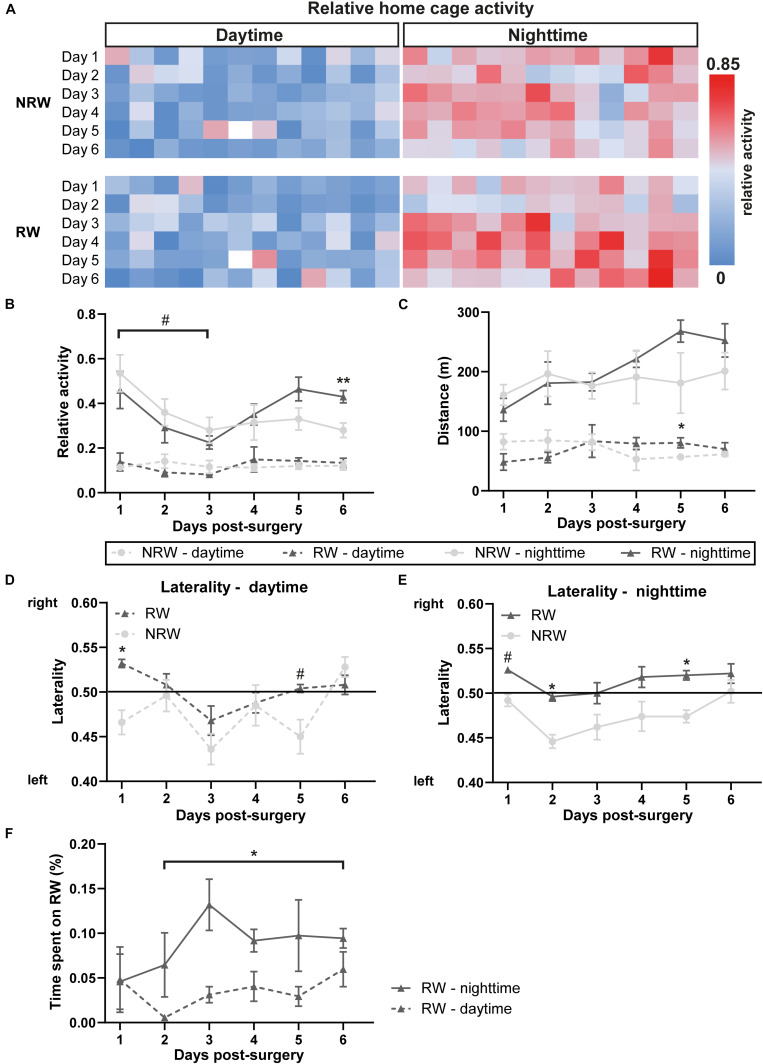
**(A)** Heatmaps showing average home cage activity per hour in RW and NRW mice recorded during 6 days post-surgery. Mice were exposed to a 12 h light cycle. DVC activity metrices were measured in RW and NRW mice during 1 week post-stroke, including **(B)** relative home-cage activity (day- and nighttime), **(C)** walked distance inside the home-cage (day- and nighttime), **(D)** daytime and **(E)** nighttime laterality index, and **(F)** the relative time spent on the running wheel. RW: *N* = 5, NRW: *N* = 5; values represented in mean ± SEM. ^#^0.05 < *p* < 0.08 (tendency); *p ≤ 0.05; **p ≤ 0.01.

The relative home-activity inside the DVC tended to decrease during the first 3 days after stroke induction during nighttime (Figure 3B: *F*(5,40) = 4.6, *p* < 0.065). Notably, 6 days after stroke an exercise effect was observed on activity during nighttime. RW mice were more active than NRW mice (Figure 3B: *F*(1,9) = 11.3, *p* < 0.010). Global home-cage activity of both NRW and RW mice per day- and nighttime during 6 days post-stroke is represented in a heatmap (Figure 3A). Furthermore, RW animals walked longer distances compared to NRW mice during daytime at 5 days post-surgery (Figure 3C: *F*(1,8) = 5.5, *p* < 0.047). No differences were found regarding the walking velocity (Supplementary Figure 3). Motor disabilities after stroke are recognized as a turning bias (laterality). During day- and nighttime 1 day post-stroke, NRW mice had a higher preference to turn left than RW animals (Figures 3D,E: daytime: *F*(1,7) = 6.8, *p* < 0.035; nighttime: *F*(1,7) = 4.5, *p* < 0.068), while this side-preference was not visible in RW mice. Likewise, the same turning preference was detected again only in NRW mice on day 5 during day- and nighttime (Figures 3D,E: daytime: *F*(1,7) = 5.5, *p* < 0.052; nighttime: *F*(1,7) = 65.5, *p* < 0.047). Only during nighttime 2 days post-stroke, NRW animals also exhibited more turns to the left side when compared to RW mice (Figure 3F: *F*(1,7) = 6.2, *p* < 0.037). The relative time mice spent in the running wheel significantly increased from day 2 to 6 during nighttime (Figure 3F: *F*(5,20) = 1.6, *p* < 0.025).

### Open Field

**FIGURE 4:**
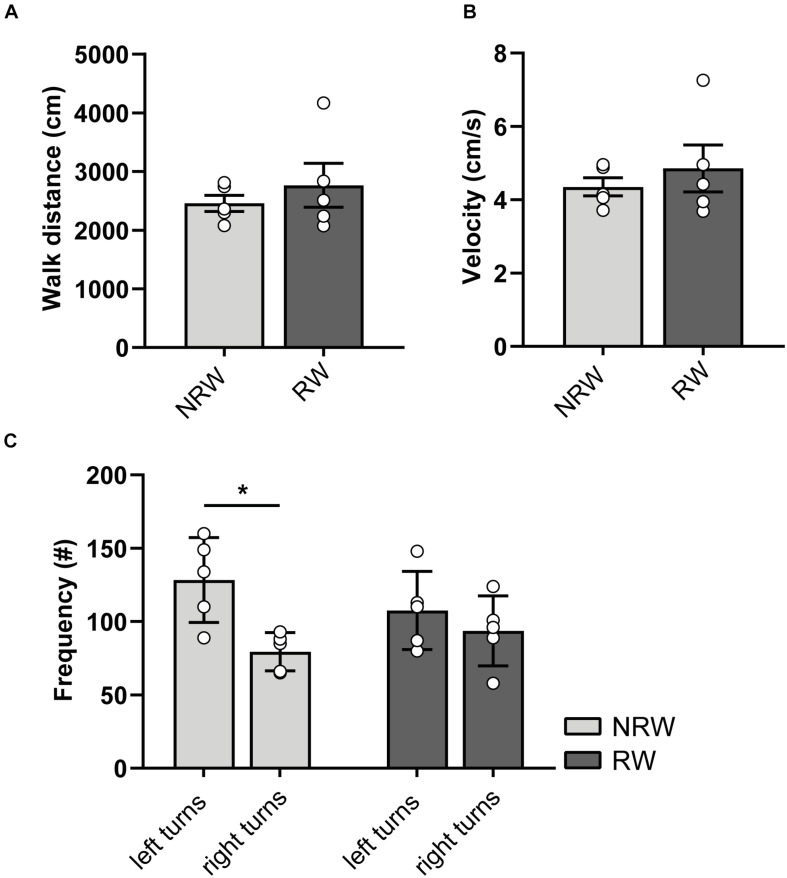
**(A)** Distance and **(B)** velocity determined during 10 min open field performed 6 days after surgery. The total distance traveled and velocity did not differ between the experimental groups. **(C)** Turning direction frequency, measured in RW and NRW animals during the open field. RW: *N* = 5, NRW: *N* = 5; values represented in mean ± SEM. *p ≤ 0.05.

At 6 days post-stroke, locomotion (distance, velocity), turning preferences, duration/frequencies in zones (Supplementary Figures 4A,B), and duration/frequency of exploratory behaviors (Supplementary Figures 4C,D) was investigated with the open field test. Notably, NRW mice showed higher preference for left side turnings (Figure 4C: *F*(1,4) = 8.0, *p* < 0.048). In accordance to the DVC data, RW mice demonstrated no turning preference (Figure 4C). No changes in behavior (Supplementary Figures 4A,B: duration/frequency in zones and Supplementary Figures 4A,B: exploratory behaviors) and locomotion (distance, velocity) (Figures 4A,B) were observed.

### Magnetic Resonance Imaging

#### Cerebral Blood Flow

**FIGURE 5:**
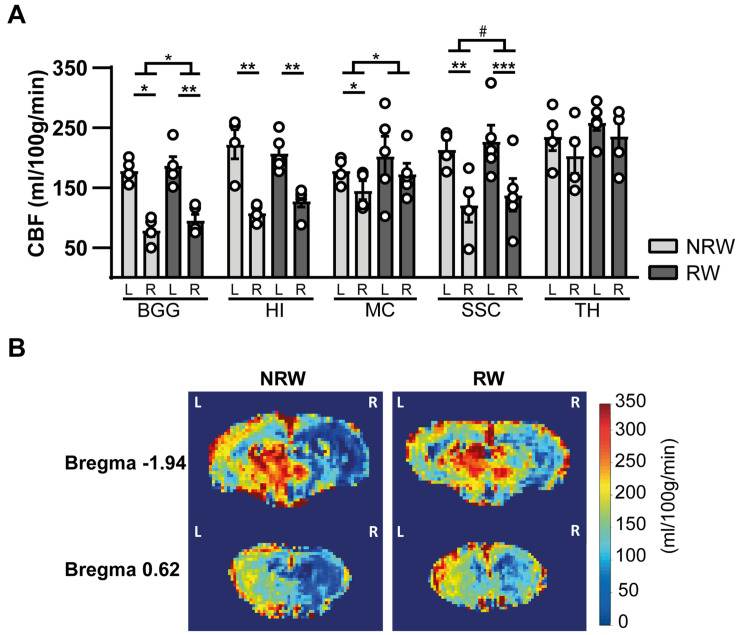
**(A)** Cerebral blood flow (CBF) 6 days after stroke induction assessed in different ROI (BGG: Basal ganglia, HI: Hippocampus, MC: Motor cortex, SSC: Somatosensory cortex) of running wheel (RW) and no running wheel (NRW) mice. **(B)** Representative high-resolution voxel-wise CBF images of two different bregma’s (–1.94 and 0.62) that were assessed in the NRW and RW group. RW: *N* = 5, NRW: *N* = 4; values represented in mean ± SEM. ^#^0.05 < *p* < 0.08 (tendency); *p ≤ 0.05; **p ≤ 0.01; ***p ≤ 0.001.

Cerebral blood flow (CBF) was determined in different ROI. CBF in motor cortex (*F*(1,7) = 7.9, *p* < 0.026) and basal ganglia (*F*(1,7) = 5.8, *p* < 0.047) was significantly higher in the RW group compared to NRW animals (Figure 5A). In the somatosensory cortex, RW mice also showed higher CBF than NRW mice (Figure 5A: *F*(1,7) = 5.2, *p* < 0.057). Several inter-hemispheric differences between the stroke affected (ipsilateral) and control (contralateral) hemisphere were measured. Both groups showed a decreased CBF in the ipsilateral hippocampus (NRW: *F*(1,3) = 43.5, *p* < 0.007, RW: (*F*(1,4) = 58.7, *p* < 0.002), somatosensory cortex (NRW: *F*(1,3) = 14.0, *p* < 0.033, RW: *F*(1,4) = 69.3, *p* < 0.001), and basal ganglia (NRW: *F*(1,3) = 33.8, *p* < 0.010; RW: *F*(1,4) = 95.7, *p* < 0.001) when compared to the contralateral brain hemisphere (Figure 5A). Moreover, only NRW animals displayed a decreased CBF in the ipsilateral motor cortex compared to corresponding contralateral brain hemisphere (Figure 5A: *F*(1,3) = 26.2, *p* < 0.014). Representative high-resolution voxel-wise CBF images are shown in Figure 5B.

#### Resting State fMRI

**FIGURE 6:**
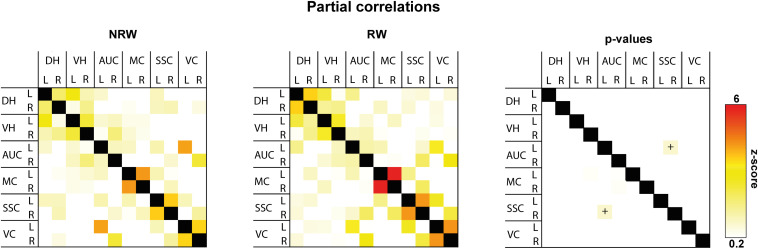
Resting-state fMRI. Partial correlations between ROI (DH: dorsal hippocampus, VH: ventral hippocampus, AUC: auditory cortex, MC: motor cortex, SSC: somatosensory cortex, VC: visual cortex) were divided in ipsilateral (R: right) and contralateral (L: left) hemisphere of running wheel (RW) and no running wheel (NRW) mice. RW: *N* = 5, NRW: *N* = 4.

Total and partial correlations of rsfMRI were assessed to analyze the functional connectivity between several ROI in the brain. With total correlations no significant effects were measured (Supplementary Figure 5). Partial correlation analysis accentuates the direct connectivity between two ROI, while regressing the temporal BOLD signal from all other ROI. RW mice displayed a higher functional connectivity between the stroke affected somatosensory cortex and control auditory cortex than NRW mice (Figure 6: *F*(1,7) = 8.5, *p* < 0.023).

### DTI

#### Fractional Anisotropy

**FIGURE 7:**
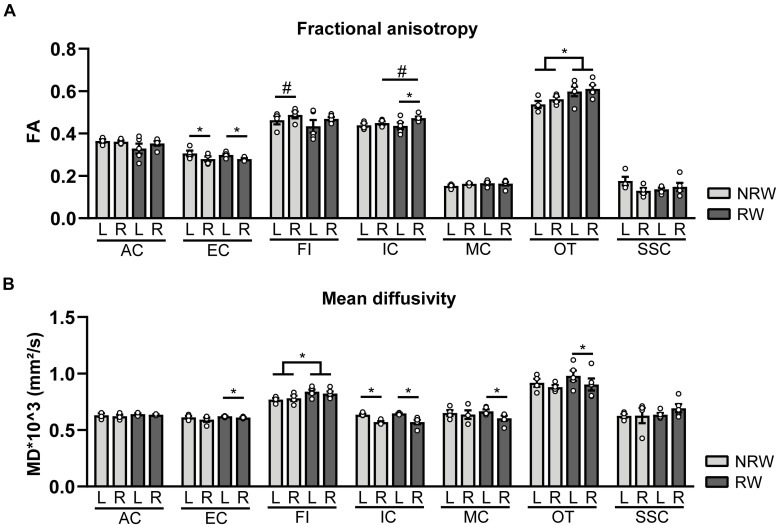
Diffusion tensor imaging (DTI) measures assessed at 1 week post-surgery in running wheel (RW) and no running wheel (NRW) animals. **(A)** Fractional anisotropy (FA) and **(B)** mean diffusivity (MD) were assessed in different ROI (AC: anterior commissure, EC: external capsule, FI: fimbria, IC: internal capsule, MC: motor cortex, OT: optic tract, SSC: somatosensory cortex) in the ipsilateral (R: right) contralateral (L: left) hemisphere. RW: *N* = 5, NRW: *N* = 4; values represented in mean ± SEM. ^#^0.05 < *p* < 0.08 (tendency); *p ≤ 0.05.

Fractional anisotropy is a measure for myelination and fiber density of WM. Elevated FA values were measured in the optic tract of the RW group when compared to the NRW mice (Figure 7A: *F*(1,7) = 6.2, *p* < 0.042). Likewise, the stroke affected (ipsilateral) internal capsule of the RW mice tended to exhibit higher FA compared to the internal capsule of NRW mice (Figure 7A: *F*(1,7) = 4.3, *p* < 0.078). Moreover, inter-hemispheric differences were detected. In both groups, FA was decreased in the ipsilateral external capsule compared to the control (contralateral) external capsule (Figure 7A: NRW: *F*(1,3) = 22.3, *p* < 0.018; RW: *F*(1,4) = 20.1, *p* < 0.011). The ipsilateral internal capsule of the RW mice exhibited higher FA than the contralateral internal capsule (Figure 7A: *F*(1,3) = 13.6, *p* < 0.035). Moreover, the ipsilateral fimbria (Figure 7A: *F*(1,3) = 7.6, *p* < 0.070), auditory cortex, and hippocampus of only RW mice tended to show higher FA than the contralateral fimbria. Only NRW animals showed lower FA in the ipsilateral auditory cortex (Supplementary Figure 6A: *F*(1,3) = 17.6, *p* < 0.025) and a tendency for lower FA in the ipsilateral hippocampus in comparison to the corresponding contralateral hippocampus (Supplementary Figure 6A: *F*(1,3) = 7.6, *p* < 0.071).

#### Mean Diffusivity

MD is the inverse measure of membrane density and sensitive measure for edema, and necrosis of GM (Le Bihan et al., 2001; Feldman et al., 2010; Alexander et al., 2011). RW mice exhibited higher MD in the fimbria than NRW mice (Figure 7B: *F*(1,7) = 5.8, *p* < 0.046). In all mice MD was significantly decreased in the basal ganglia (Supplementary Figure 6B: NRW: *F*(1,3) = 24.7, *p* < 0.016; RW: *F*(1,4) = 25.5, *p* < 0.007), hippocampus (Supplementary Figure 6B: NRW: *F*(1,3) = 11.9, *p* < 0.041; RW: *F*(1,4) = 9.1, *p* < 0.039), and internal capsule (Figure 7B: NRW: *F*(1,3) = 26.4, *p* < 0.014; RW: *F*(1,4) = 20.8, *p* < 0.010) of the ipsilateral hemisphere when compared to the contralateral hemisphere. A trend in the same direction was found in the amygdala in both experimental groups (Supplementary Figure 6B: NRW: *F*(1,3) = 9.3, *p* < 0.055; RW: *F*(1,4) = 5.9, *p* < 0.072). Furthermore, only RW mice exhibited a decreased MD in the ipsilateral external capsule (Figure 7B: *F*(1,4) = 7.9, *p* < 0.048), motor cortex (Figure 7B: *F*(1,4) = 11.9, *p* < 0.026), optic tract (Figure 7B: *F*(1,4) = 11.3, *p* < 0.028), and a tendency for lower MD levels in the ipsilateral forceps minor (Supplementary Figure 6B: *F*(1,4) = 6.5, *p* < 0.063) when compared to the corresponding contralateral ROI (Figure 7B). No differences in FA and MD were detected in other ROI (Supplementary Figure 6).

### Post-mortem Analysis

#### Polarized Light Imaging

##### Retardance

**FIGURE 8:**
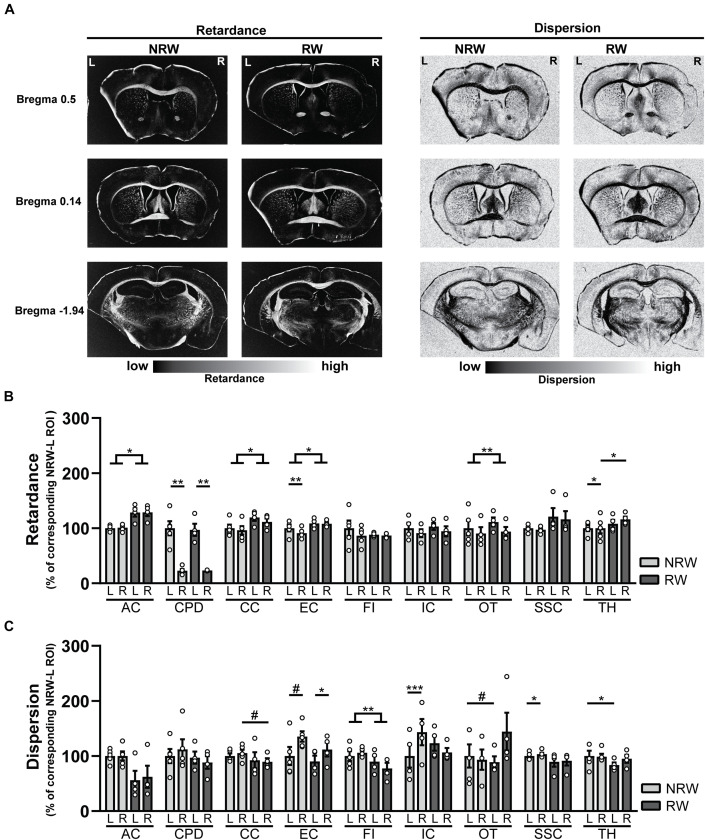
**(A)** Representative images of retardance and dispersion maps of both running wheel (RW) and no running wheel mice (NRW) 1 week post-stroke, taken at the level of bregma 0.14, 0.5, and –1.94. **(B)** Retardance and **(C)** dispersion assessed with polarized light imaging (PLI) in the ipsilateral (R: right) contralateral (L: left) hemisphere of several ROI (AC: anterior commissure, CPD: cerebral peduncle, CC: corpus callosum, EC: external capsule, FI: fimbria, HI: hippocampus, IC: internal capsule, MC: motor cortex, OT: optic tract, SSC: somatosensory cortex, TH: thalamus). RW: *N* = 5, NRW: *N* = 4; values represented in mean ± SEM. ^#^0.05 < *p* < 0.08 (tendency); *p ≤ 0.05; **p ≤ 0.01; ***p ≤ 0.001.

Decreased retardance values can be used as an indirect measurement for myelin loss. Several exercise effects were revealed, showing higher retardance in RW mice compared to NRW animals. RW mice displayed higher retardance in the anterior commissure (*F*(1,5) = 11.3, *p* < 0.020), corpus callosum (*F*(1,5) = 7.1, *p* < 0.044), external capsule (*F*(1,5) = 6.8, *p* < 0.047) and optic tract (*F*(1,5) = 29.4, *p* < 0.003) than NRW mice (Figure 8B). In addition, higher retardance was measured in the ipsilateral thalamus among RW mice when compared to the ipsilateral thalamus in NRW mice (*F*(1,5) = 11.4, *p* < 0.020). Stroke related inter-hemispheric differences were detected in the NRW group, showing decreased retardance in the stroke affected (ipsilateral) brain hemisphere of the external capsule (*F*(1,4) = 10.0, *p* < 0.034) and thalamus (*F*(1,4) = 14.0, *p* < 0.033) when compared to the control (contralateral) side of the respective ROI (Figure 8B). Likewise, the retardance in the ipsilateral cerebral peduncle was lower when compared to the contralateral hemisphere in both groups (Figure 8B: NRW: *F*(1,4) = 25.9; *p* < 0.007; RW: *F*(1,5) = 42.8, *p* < 0.007).

##### Dispersion

In PLI the calculation of dispersion provides a quantitative estimate of fiber dispersal (Mollink et al., 2017). Decreased dispersion values are indicative for better myelin quality. Dispersion was decreased in the fimbria among RW mice in comparison to NRW animals (Figure 8C: *F*(1,5) = 37.6, *p* < 0.002). In RW mice the ipsilateral corpus callosum (Figure 8C: *F*(1,5) = 5.8, *p* < 0.061) and the contralateral side of the optic tract (Figure 8C: *F*(1,5) = 4.8, *p* < 0.079) tended to display lower dispersion than the same corresponding ROI in NRW mice (Figure 8C). Likewise, lower dispersion was measured in the contralateral thalamus of the RW mice compared to contralateral thalamus of the NRW mice (Figure 8C: *F*(1,5) = 6.6, *p* < 0.050). Stroke-related interhemispheric differences were found in several ROI. Lower dispersion values were measured in the ipsilateral external capsule of all animals when compared to the contralateral external capsule (Figure 8C: NRW: *F*(1,4) = 5.9, *p* < 0.072; RW: (*F*(1,3) = 0.29, *p* < 0.012). In contrast, the ipsilateral internal capsule (*F*(1,2) = 2423.0, *p* < 0.001) and somatosensory cortex (*F*(1,4) = 9.2, *p* < 0.038) of only NRW animals also displayed higher dispersion than the corresponding region of the contralateral brain hemisphere (Figure 8C).

Several ROI displayed no significant differences in retardance and dispersion (Supplementary Figure 7). Representative images of dispersion and retardance maps are shown in Figure 8A.

#### IBA-1 Staining

**FIGURE 9:**
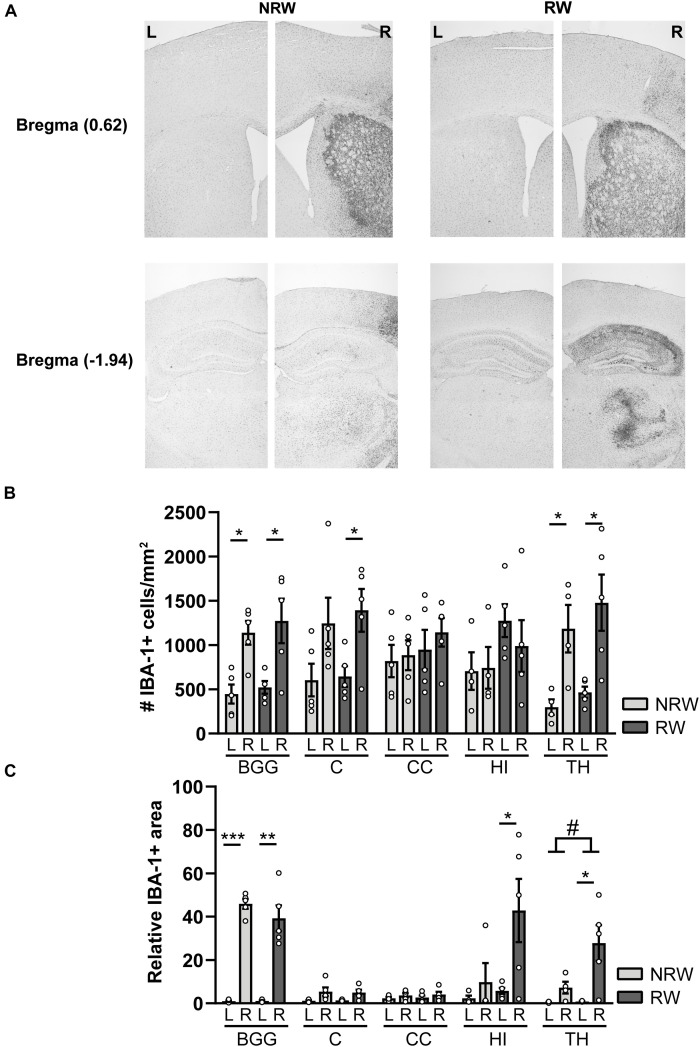
Immunohistochemical staining for IBA-1 in brains of mice with (RW) and without a running wheel (NRW) performed 1 week after stroke induction. **(A)** Representative images of the IBA-1 staining in the ipsilateral (R: right) and contralateral (L: left) hemisphere of bregma –1.94 and 0.62 in RW and NRW animals. **(B)** Number of IBA-1+ positive cells and **(C)** relative IBA-1+ area in different ROI (BGG: basal ganglia, C: cortex, CC: corpus callosum, H: hippocampus, TH: thalamus) were calculated. RW: *N* = 5, NRW: *N* = 4; values represented in mean ± SEM. ^#^0.05 < *p* < 0.08 (tendency); *p ≤ 0.05; **p ≤ 0.01; ***p ≤ 0.001.

IBA-1 is a calcium binding protein which is specifically expressed in activated microglia and macrophages in the brain. Representative images of the IBA-1 staining are displayed in Figure 9A. In this study, we assessed the amount of IBA-1+ cells and the relative IBA-1+ area in different ROI. Both experimental groups showed more IBA-1+ cells in the thalamus (NRW: *F*(1,3) = 22.5, *p* < 0.018; RW: *F*(1,4) = 9.0, *p* < 0.040) and the basal ganglia (NRW: (*F*(1,3) = 12.5, *p* < 0.024; RW: *F*(1,4) = 12.5, *p* < 0.024) of the stroke affected (ipsilateral) hemisphere in comparison to the corresponding control (contralateral) hemisphere (Figure 9B). In addition, only the RW group exhibited more IBA-1+ cells in the ipsilateral cortex when compared to the contralateral cortex (Figure 9B: *F*(1,4) = 15.5, *p* < 0.017). In line with the inter-hemispheric differences regarding the amount of IBA-1+ cells, we also measured an increased relative IBA-1+ area in the ipsilateral basal ganglia among both groups (NRW: *F*(1,3) = 435.6, *p* < 0.001; RW: *F*(1,4) = 38.4, *p* < 0.003) as well as in the ipsilateral thalamus (*F*(1,4) = 10.6, *p* < 0.031), hippocampus (*F*(1,4) = 7.7, *p* < 0.05), and cortex (*F*(1,4) = 7.5, *p* < 0.052) of the RW animals compared to the corresponding contralateral hemisphere (Figure 9C). Additionally, the thalamus of RW mice displayed a slightly increased relative IBA-1+ area compared to NRW mice (Figure 9C: *F*(1,4) = 4.8, *p* < 0.065).

#### GLUT-1 Staining

**FIGURE 10:**
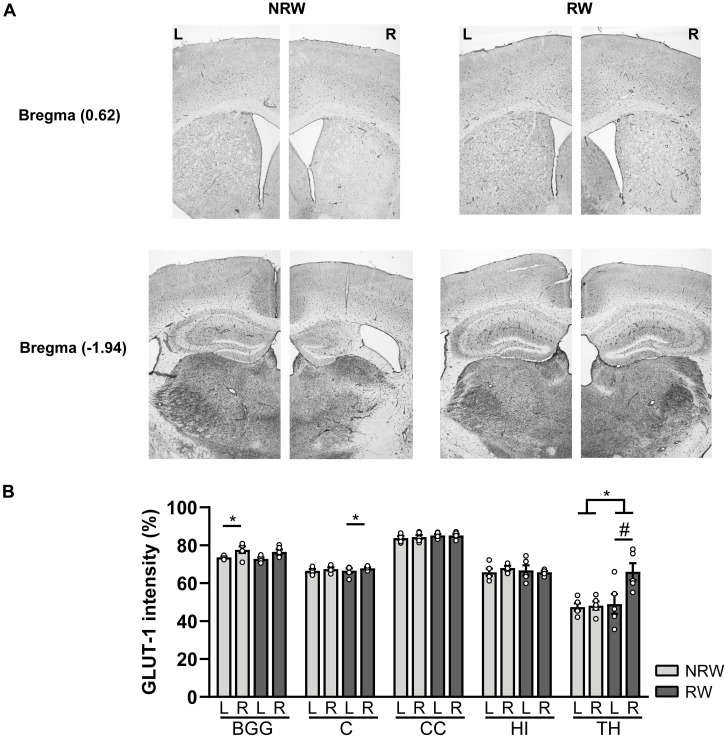
Immunohistochemical staining for GLUT-1 in brains of mice with (RW) and without a running wheel (NRW) 1 week after stroke induction. **(A)** Representative images of the GLUT-1 staining in the ipsilateral (R: right) and contralateral hemisphere (L: left) of bregma –1.94 and 0.62 in RW and NRW mice. **(B)** GLUT-1 intensity measured in different ROI (BGG: basal ganglia, C: cortex, CC: corpus callosum, H: hippocampus, TH: thalamus). RW: *N* = 5, NRW: *N* = 5; values represented in mean ± SEM. ^#^0.05 < *p* < 0.08 (tendency); *p ≤ 0.05.

A GLUT-1 immunohistochemical staining was performed to investigate the impact of voluntary exercise on changes of GLUT-1 intensity, a marker for the amount of GLUT-1 transporter. In Figure 10A representative images of the GLUT-1 staining are shown. The amount of GLUT-1 measured in the thalamus was higher in RW mice compared to NRW animals (Figure 10B: *F*(1,8) = 5.5, *p* < 0.047). Higher GLUT-1 levels were observed in the stroke affected (ipsilateral) thalamus (RW: *F*(1,4) = 10.3, *p* < 0.058), basal ganglia (RW: *F*(1,4) = 12.2, *p* < 0.025), and cortex (NRW: *F*(1,4) = 9.3, *p* < 0.038) when compared to the corresponding control (contralateral) ROI Figure 10B. Furthermore, we assessed the number of GLUT-1+ blood vessels, indicative for vascular density. Significant less GLUT-1+ blood vessels were measured in the ipsilateral thalamus of RW mice when compared to NRW mice (Supplementary Figure 8: *F*(1,8) = 5.9, *p* < 0.042). In the ipsilateral basal ganglia RW mice displayed less GLUT-1+ blood vessels than in the contralateral basal ganglia (Supplementary Figure 8: *F*(1,4) = 17.1, *p* < 0.014). Moreover, the relative GLUT-1+ area tended to be larger than in the ipsilateral hippocampus among RW mice when compared to NRW mice (Supplementary Figure 8: *F*(1,8) = 5.3, *p* < 0.051).

## Discussion

Millions of people suffer from ischemic stroke every year. The existing stroke treatments are insufficient which is reflected by high numbers of death and disability worldwide (Hankey, 2017). New insights in the therapeutic effects of exercise after ischemic stroke are available, showing benefits in restoring brain damage as well as functional rehabilitation (Luo et al., 2007; Voss et al., 2013; Zhang et al., 2013). While there is general consensus that recovery of brain connectivity (structural and functional) is crucial for functional stroke rehabilitation, studies investigating the impact of exercise on brain networks are lacking (Wu et al., 2017). Therefore, we assessed the impact of voluntary exercise on the recovery of stroke induced mice with a combination of functional and structural MRI measures (DTI, rsfMRI, CBF), behavioral parameters, molecular markers (inflammation, vascular density and quality, angiogenesis) and compared their recovery to a control group without access to running wheels. In addition to widely used white matter tractography by DTI, we quantified stroke related white matter degeneration postmortem, with the very innovative PLI technique for the first time in murine brains. In addition, home cage activity, locomotion, and walking patterns has been meticulously monitored 24/7 in DVC without disturbing the natural behavior of the animals. In addition, home cage activity, locomotion, and walking patterns has been meticulously monitored 24/7 in DVC without disturbing the natural behavior of the animals. This study demonstrates, that 6 days of voluntary exercise after ischemic stroke is already able to improve CBF, vascular quality (amount of GLUT-1 transporter), functional connectivity, as well as white matter fiber density and white matter fiber orientation in the brain. Subsequently, also motor abilities, such as turning preference, and overall activity were improved in RW mice when compared to NRW animals at day 6 post-surgery. An overview of all effects can be found in Supplementary Table 4.

### Cerebrovasculature

Restoration of the cerebral vasculature is crucial for stroke recovery (Markus, 2004). In this study, we detected a decreased CBF in several brain regions located in the ipsilateral brain hemisphere 1 week post-stroke, which is in line with our previous study (Wiesmann et al., 2017). In contrast to the NRW group, no inter-hemispheric differences could be detected in the motor cortex of RW mice. Notably, CBF in the motor cortex, somatosensory cortex and basal ganglia was higher among RW animals when compared to NRW mice. Thus, voluntary exercise can maintain increase CBF post-stroke, especially in the motor cortex.

The reduction of CBF during stroke can minimize glucose uptake by reducing the capillary surface area (vascular density) available for glucose transport (Winkler et al., 2015). The GLUT-1+ staining intensity, indicative for the amount of GLUT-1+ transporters in the capillaries, acts as a measure for vascular quality. Vascular quality was increased in the thalamus of RW mice compared to NRW animals. In addition, only in RW mice, more GLUT-1+ transporters were found in thalamus and basal ganglia of the ipsilateral hemisphere in comparison to the respective contralateral hemisphere. Increased vascular quality in the right basal ganglia is consistent with the finding of increased CBF in the basal ganglia among RW mice. The vascular density (number of GLUT-1+ blood vessels) was decreased among RW mice in the ipsilateral thalamus. These improvements in vascular quality were also partially in line with CBF results, suggesting better glucose and oxygen uptake among RW mice (Winkler et al., 2015).

Moreover, RW mice tended to express higher VEGF mRNA levels. VEGF is pivotal for stroke recovery by promoting angiogenesis and neurogenesis in the penumbra (Sun et al., 2003). In accordance, a preclinical study has shown that short-term treadmill exercise (3 days) can elevate the VEGF expression levels in the brain (Ma et al., 2013). Exercise-induced vascular remodeling, induced by elevated VEGF expression, may result in better vascular density which in turn promotes the restauration of CBF in the stroke area.

### Brain Structure and Function

The extend of WM degeneration is crucial for functional outcome in stroke patients, rather than only stroke size (Zhang et al., 2018). Therefore, we assessed *in vivo* DTI as a neuroimaging biomarker for WM and GM integrity 1 week after tMCAO in the same and additional ROI that were used for CBF analysis. WM degeneration after ischemic stroke is associated with reduced fractional anisotropy (FA) and increased mean diffusivity (MD) (Wiesmann et al., 2017, 2018; Pinter et al., 2020).

Impact of stroke was reflected by lower FA in several ROI located in the ipsilateral hemisphere compared to corresponding ROI in the contralateral hemisphere. In contrast, stroke-related decrease in FA was only observed in the ipsilateral auditory cortex and hippocampus of NRW mice, but not in RW animals. Interestingly, RW animals displayed higher FA values in the optic tract and in the ipsilateral internal capsule compared to NRW mice. In contrast, no impact of exercise could be detected in ROI located in GM. Previous research has shown that aerobic excise positively correlates with FA values measured with DTI in healthy individuals/animals (Johnson et al., 2012; Chaddock-Heyman et al., 2018; Islam et al., 2020). Our study is the first DTI study that recapitulates the positive effects of voluntary exercise on WM fiber integrity in stroke induced mice. A recent animal study demonstrated increased numbers of WM oligodendrocyte precursor cells in an animal model of ischemic vascular dementia after treadmill exercise. It has to be investigated if causality exists between exercise induced increase in FA and remyelination via oligodendrocyte precursor cells post-surgery (Ohtomo et al., 2019).

After stroke, increased MD is a marker for the presence of edema, cellularity, and necrosis in GM (Le Bihan et al., 2001; Feldman et al., 2010; Alexander et al., 2011). In contrast, we found lower MD values in the ipsilateral brain hemispheres of several ROI when compared to the corresponding contralateral hemispheres, independent of the experimental group. Evident MD reduction is generally seen in the infarct area immediately after stroke onset (Zhang et al., 2018). It can be hypothesized that decreased MD in ipsilateral brain regions were caused by persistent edema 1 week post-stroke. One pitfall of *in vivo* DTI is that the spatial resolution is much lower than equivalent estimates derived from microscopy data (Mollink et al., 2017).

In addition to *in vivo* DTI parameters, we performed *postmortem* PLI microscopy. PLI is a novel technique to investigate the orientation of myelinated fibers, based on the birefringence property of myelin (Axer et al., 2011; Mollink et al., 2017). To our knowledge, this is the first study that makes use of PLI to investigate WM degeneration in murine brain tissue. PLI retardance values are representative for myelination and corresponds to FA in DTI, whereas PLI dispersion is a value of fiber orientation, comparable to MD in DTI (Mollink et al., 2017).

PLI retardance data provide consistent evidence that exercising is beneficial for the recovery of myelin density after stroke. In several regions in the WM, RW mice displayed significant higher retardance than NRW animals. This finding indicates that RW mice had higher myelin density than NRW mice 1 week post-stroke. It can be hypothesized that voluntary exercise either prevented demyelination or promotes remyelination after stroke. Studies have shown that exercise can significantly reduce demyelination after stroke (Ahn et al., 2016; Cheng et al., 2020). However, the destructive effects of stroke on myelination were still observable as inter-hemispheric differences in both experimental groups.

Moreover, PLI dispersion values indicated signs of impaired WM integrity among NRW mice, but not in RW animals (Mollink et al., 2017). In brain regions of NRW mice increased dispersion values were measured when compared to the corresponding ROI in RW animals, suggesting WM degeneration. In contrast, WM degeneration was not recapitulated by increased MD values in this study. It can be hypothesized that edema might have caused decreased MD values in NRW animals, although microstructural changes were already established (Umarova et al., 2017). In conclusion, PLI measures revealed that voluntary exercise showed a positive effect on both WM density and integrity. Minor voluntary exercise induced differences have been detected in less myelinated GM regions. Overall, PLI dispersion values did not match the direction of MD values. Considering that this is the first study investigating myelin degeneration with PLI in stroke-induced murine brains, more research is needed to evaluate the impact of voluntary exercise on both, DTI and PLI parameters.

Furthermore, neurological damage after stroke results in loss of functionality and connectivity of brain networks that can be measured with rsfMRI in mice (Jonckers et al., 2015). In our previous study, we showed that stroke decreases functional connectivity in mice (Calahorra et al., 2019). Up to now, no animal studies have been performed investigating the impact of exercise on functional connectivity. Studies in human stroke patients indicate that functional post-stroke brain reorganization is associated with preserved functional connectivity in motor to nonmotor networks (Almeida et al., 2017). In the current study, partial correlations of functional connectivity data showed a positive effect of voluntary exercise already 1 week post-stroke. The RW group showed increased functional connectivity between the ipsilateral somatosensory cortex and contralateral auditory cortex compared to NRW mice. Considering the high importance of intact WM integrity post-stroke, current findings indicate that voluntary exercise has neuroprotective effects on WM which in turn also facilitates recovery of functional connectivity in the brain.

### Activity and Motor Function

One of the most common symptoms, recognized in patients after an ischemic event, includes persistent motor deficits (Hatem et al., 2016). In the present study, we showed an increase in activity and motor skills in animals with access to voluntary exercise for 1 week post-stroke. Previously, we found that stroke induced mice, but not sham animals, display decreased home-cage activity in the 1st week after stroke (Calahorra et al., 2019). In accordance, we also found that activity was decreasing in the 1st days after stroke induction in RW and NRW mice. Nighttime activity was increased in the RW group 6 days after stroke surgery in comparison to NRW mice, indicating that only stroke operated RW mice recover to the activity level of sham operated mice 1 week post-stroke. Only 5 days post-stroke, RW mice also walked longer distances in their home-cage during daytime. After stroke induction in the right brain hemisphere, animals develop a turning preference towards the left side due to motor deficits induced by ischemic damage (Calahorra et al., 2019; Shenk et al., 2020). During the open field test, NRW animals exhibited a preference for left turns, while RW animals did not exhibit a turning bias. In accordance, the laterality index, based on DVC home cage activity measurements, reflected also that NRW had higher preference to turn to the left side than RW mice. No DVC laterality data prior surgery are available due to group-housing, however, we found previously that a turning bias measured by DVC was exclusively detected post-stroke (Calahorra et al., 2019). The improvements in motor skills and activity found in RW animals were most likely facilitated by improved CBF and vascular quality in motor regions of the brain, increased functional connectivity, and enhanced structural integrity of WM. In a recent study, improved recovery of motor function has been found in stroke patients following a forced exercise paradigm (Hatem et al., 2016). Motor recovery has been proven to be dependent on functional and structural brain network rehabilitation (Guggisberg et al., 2019). The presented data provide evidence that voluntary exercise can support this mechanism and helps to counteract stroke-related motor impairments.

### Molecular Alterations

After stroke the injured area attracts immune cells which can exacerbate the lesions (Otxoa-de-Amezaga et al., 2019). Acute post-stroke, inflammation is crucial for clearance of apoptotic tissue and restoration of neuronal function, while excessive inflammatory responses can aggravate stroke outcomes. We found no significant exercise effect on the amount of IBA-1 positive cells and the infarct size. Only in the thalamus relative IBA+ area tended to be increased in RW mice in comparison to NRW mice. Inter-hemispheric differences were detected in both experimental groups in the cortex, basal ganglia, hippocampus and thalamus, which reflected increased inflammation following an ischemic stroke (Wiesmann et al., 2017, 2018). The results suggest that voluntary exercise had slight immunomodulatory properties 6 days post-stroke. There is evidence that voluntary exercise modulates microglia/macrophages activation in stroke induced mice during 10 weeks after MCAO surgery (Kohman et al., 2013). It needs to be further elucidated whether voluntary exercise promotes or exacerbates brain recovery after stroke by investigating distinct microglia polarization markers (anti- vs. pro-inflammatory) with appropriate techniques (scRNA and spatial transcriptomics).

## Conclusion

In this study, we have shown in 3 months old stroke mice, resembling mature adult humans (Flurkey and Harrison, 2007), that even short-term (1 week) voluntary exercise positively influenced the recovery in experimental stroke mice. Since the presented short-term voluntary exercise paradigm already resulted in promising therapeutic effects, we suggest to further elucidate the impact of voluntary exercise on stroke recovery longitudinally, including additional motor and behavioral tests (e.g., Bederson score, elevated body swing test). In addition, to develop tailor made stroke rehabilitation programs, the efficacy of voluntary exercise also needs to be further investigated in mice of both genders as well as in aged mice. In particular, it is important to perform experiments in female mice, since the severity of stroke-induced brain damage and treatment effects of exercise might differ due to estrogen (Roy-O’Reilly and McCullough, 2018). Overall, long-term regular exercise combined with healthy diet, should be implemented in treatment programs of stroke survivors to achieve better recovery. Exercise should be especially implemented in the rehabilitation programs of (young) stroke patients that do not suffer from severe disabilities. Stroke patients with severe disabilities should start with moderate physiotherapy as soon as possible.

## Data Availability Statement

The datasets generated for this study are available on request to the corresponding author.

## Ethics Statement

The animal study was reviewed and approved by the Dutch federal law for animal experimentation (“Wet op de Dierproeven”, 1996), the regulations of the European Union Directive of 22 September 2010 (2010/63/EU), and the Animal Ethics Committee of the Radboudumc (protocol number: 2015-0079-006 RU-DEC).

## Author Contributions

AK and MW provided conceptual guidance and were together with KL involved in the study design. KL, MW, and VV collected all the experimental data. JS performed data analysis with Traja. KL performed statistical analysis. The manuscript was written by KL and commented on by all authors.

## Conflict of Interest

The authors declare that the research was conducted in the absence of any commercial or financial relationships that could be construed as a potential conflict of interest.
