# Supplementary material for: The Impact of Voluntary Exercise on Stroke Recovery
Source: Front Neurosci. 2021 Jul 12;15:695138. doi: 10.3389/fnins.2021.695138 (PMC8311567; doi:10.3389/fnins.2021.695138)
Supplement: Supplementary file 1 [file Data_Sheet_1.docx]

Supplementary Material

**Results**

**Supplementary Table 1** Excluded mice per experiment. One mouse was excluded from the analysis, because MRI scans showed e.g. movement-, EPI-related artifacts. Furthermore, one mouse was excluded from immunohistochemical staining and PLI, because of severe tissue damage (stroke hemisphere).

| Experiment | No running wheel | Running wheel |
| --- | --- | --- |
| Open field | 0 | 0 |
| Digital ventilated cages | 0 | 0 |
| MRI – Stroke size | 1 | 0 |
| MRI – Arterial spin labelling | 1 | 0 |
| MRI – Diffusion tensor imaging | 1 | 0 |
| Resting state functional MRI | 1 | 0 |
| qPCR | 0 | 0 |
| IBA-1 staining | 1 | 0 |
| GLUT-1 staining | 0 | 0 |
| Polarized light imaging – Dispersion | 1 | 0 |
| Polarized light imaging – Retardance | 1 | 0 |


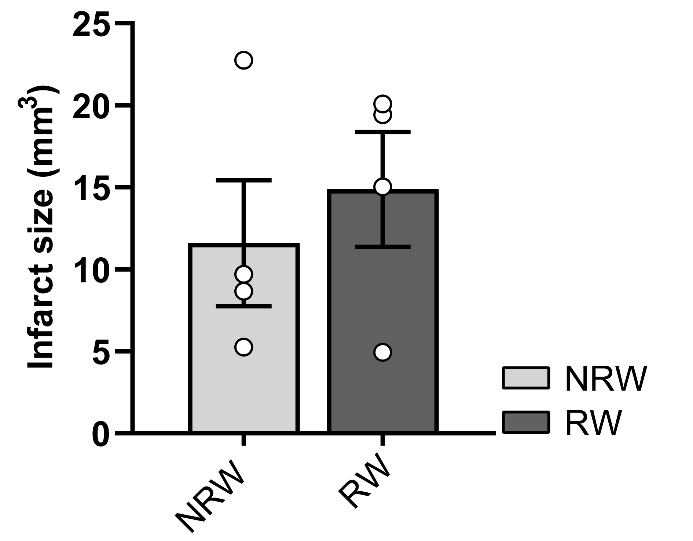


**Supplementary Figure 1** Infarct size was similar among running wheel (RW) and no running wheel (NRW) mice at day 6 after surgery (F(1,7)=0.27, p<0.62). RW: N=5, NRW: N=4; values represented in mean ± SEM.

**Supplementary Table 2** Imaging sequences for the anatomical references, rsfMRI, ASL, DTI, stroke RARE and Stoke DWI.

|  | Imaging method | Echo time (ms) | Repetition time | Image matrix | Field-of-view (mm) | Spatial resolution (μm/pixel) | No. of slices | Total acquisition time (min) |
| --- | --- | --- | --- | --- | --- | --- | --- | --- |
| Anatomical T2*w | GE | 7.357 | 865.086 ms | 512 × 512 | 40 × 40 | 78 × 78 × 500 | 20 × 3 | ∼8 |
| rsfMRI | Spin-echo EPI | 10 | 1.8 s | 96 × 96 | 25 × 25 | 260 × 260 × 500 | 20 | ∼11 |
| CBF | FAIR-ASL | 10.08 | 12 s | 128 × 128 | 25 × 25 | 260 × 260 × 1000 | 1 | ∼13 |
| Diffusion tensor imaging | 6-shot spin-echo EPI | 21 | 7.75 s | 128 × 128 | 20 × 20 | 156 × 156 × 500 | 20 | ∼35 |
| Stroke RARE | RARE | 40 | 2.5 s | 512 × 512 | 25.6 × 25.6 | 50 × 50 × 500 | 12 | ∼4 |
| Stroke DWI | Spin-echo DW_Contrast | 27 | 2.25 s | 128 × 128 | 12.8 × 12.8 | 100 × 100 × 500 | 9 | ∼15 |

**Supplementary Table 3** List of PCR primers that were used. We assessed gene expression of Vascular endothelial growth factor (Vegf), brain derived neurotrophic factor (BDNF), glucose transporter 1 (GLUT-1), postsynaptic density protein 95 (Psd95), beta-2 microglobulin (B2M), Synaptophysin, and Glyceraldehyde 3-phosphate dehydrogenase (GAPDH). In RW mice Vegf expression was slightly higher than in NRW mice (Fig. S1: F(1,6)=5.0, p<0.068).

| Gene | Direction | Sequence (5’ to 3’) |
| --- | --- | --- |
| Vegf | Forward | CAAGATCCGCAGACGTGTAA |
|  | Reverse | CGCCTTGGCTTGTCACAT |
| GLUT-1 | Forward | GATCCCAGCAGCAAGAAGGT |
|  | Reverse | TAGCCGAACTGCAGTGATCC |
| Psd95 | Forward | TGGATCACAGGGTCGAGAAGA |
|  | Reverse | TTGGCACGGTCTTTGGTAGG |
| Synaptophysin | Forward | GAGTGTGCCAACAAGACGGA |
|  | Reverse | CACTTGGTGCAGCCTGAATG |
| B2M | Forward | GATGTCAGATATGTCCTTCAGCA |
|  | Reverse | TCACATGTCTCGATCCCAGT |
| GAPDH | Forward | GTCGGTGTGAACGGATTTGG |
|  | Reverse | ACAATCTCCACTTTGCCACTG |


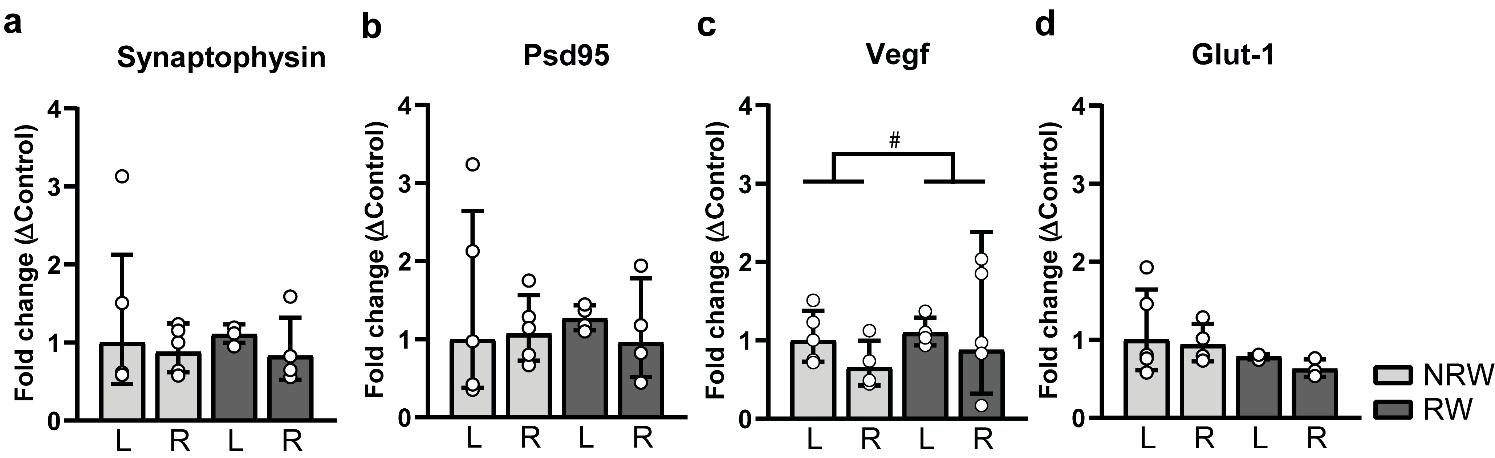
 **Supplementary Figure 2** Relative RNA expression of Syp, Psd-59, Vegf, and Glut-1 in running wheel (RW) and no running wheel animals (NRW). (A, B, D) No significant differences were found in Syp, Psd-59, Vegf, and Glut-1 expression. (A) A tendency for higher Vegf expression was found in RW mice when compared to NRW mice (F(1,6)=5.0, p<0.068). RW: N=5, NRW: N=5; values represented in geometric mean ± geometric SD. #, 0.05 < p < 0.08 (tendency).


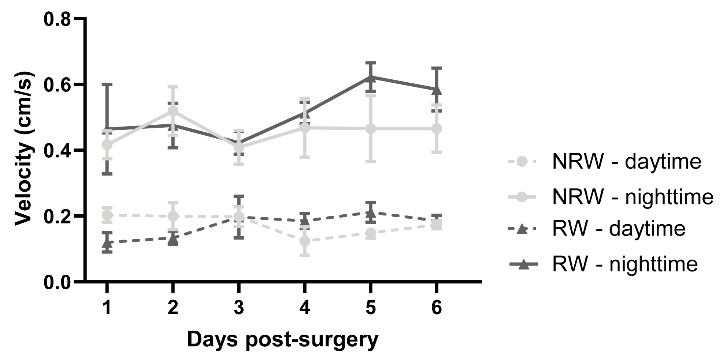


**Supplementary Figure 3** DVC activity metrices. Velocity measured in digital ventilated cages during day- and nighttime for 6 days after stroke surgery. No significant differences between running wheel (RW) and no running wheel (NRW) mice have been measured. RW: N=5, NRW: N=5; values represented in mean ± SEM.


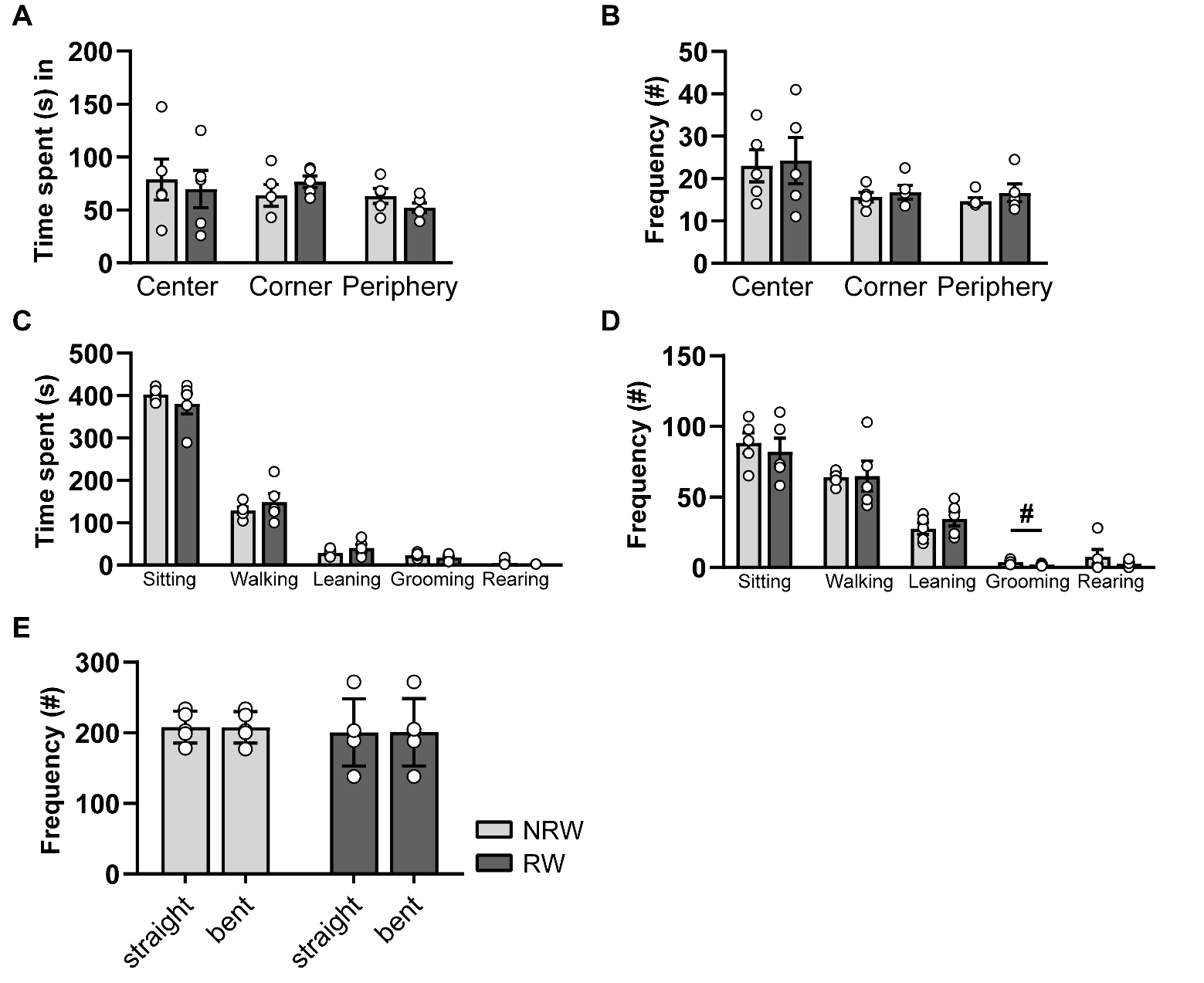


**Supplementary Figure 4** Open field 6 days after stroke induction in running wheel (RW) and no running wheel (NRW) mice. No significant differences were found regarding (A) the time animals spent in zones (center, corner, periphery), (B) the frequency animals entered zones (center, corner, periphery), (C) the time animals spent during manually scored activities (sitting, walking, leaning, grooming, rearing), and (D) the frequency mice had a bent or straight body angle. (E) RW mice tended to groom less often than NRW mice. RW: N=5, NRW: N=5; values represented in mean ± SEM #, 0.05 < p < 0.08 (tendency).


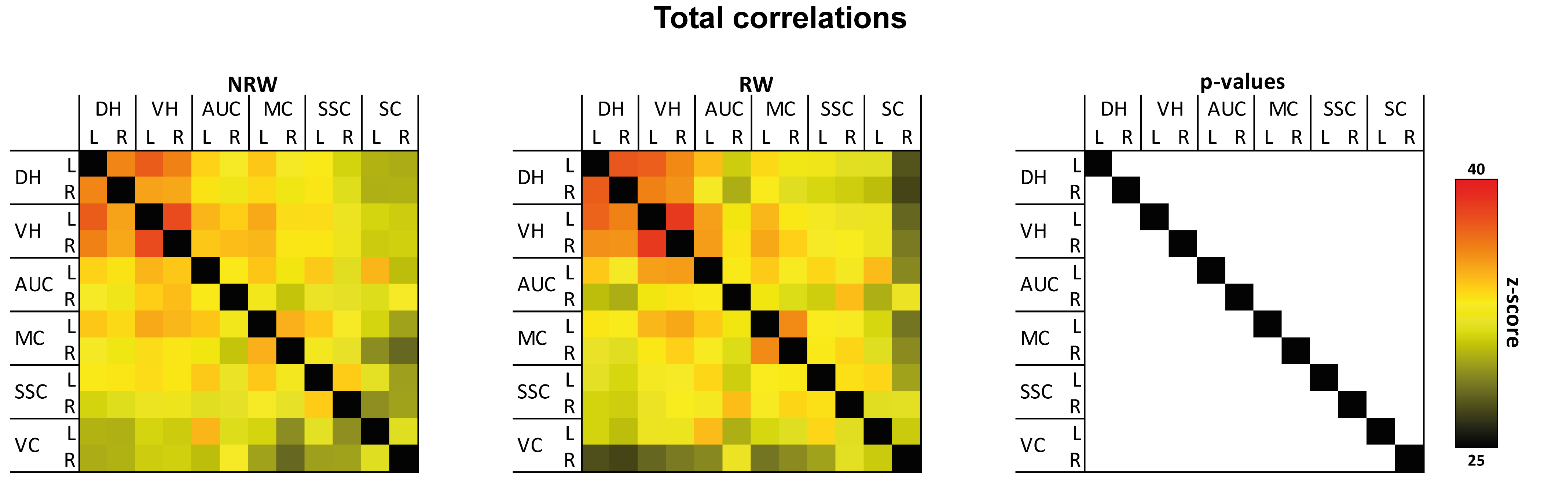


**Supplementary Figure 5** Resting state fMRI. Total correlations between regions of interest (DH: dorsal hippocampus, VH: ventral hippocampus, AUC: auditory cortex, MC: motor cortex, SSC: somatosensory cortex, VC: visual cortex) were divided in ipsilateral (R: right) and contralateral (L: left) hemisphere of running wheel (RW) and no running wheel (RW) group. No significant differences were found. RW: N=5, NRW: N=4; values represented in mean ± SEM.


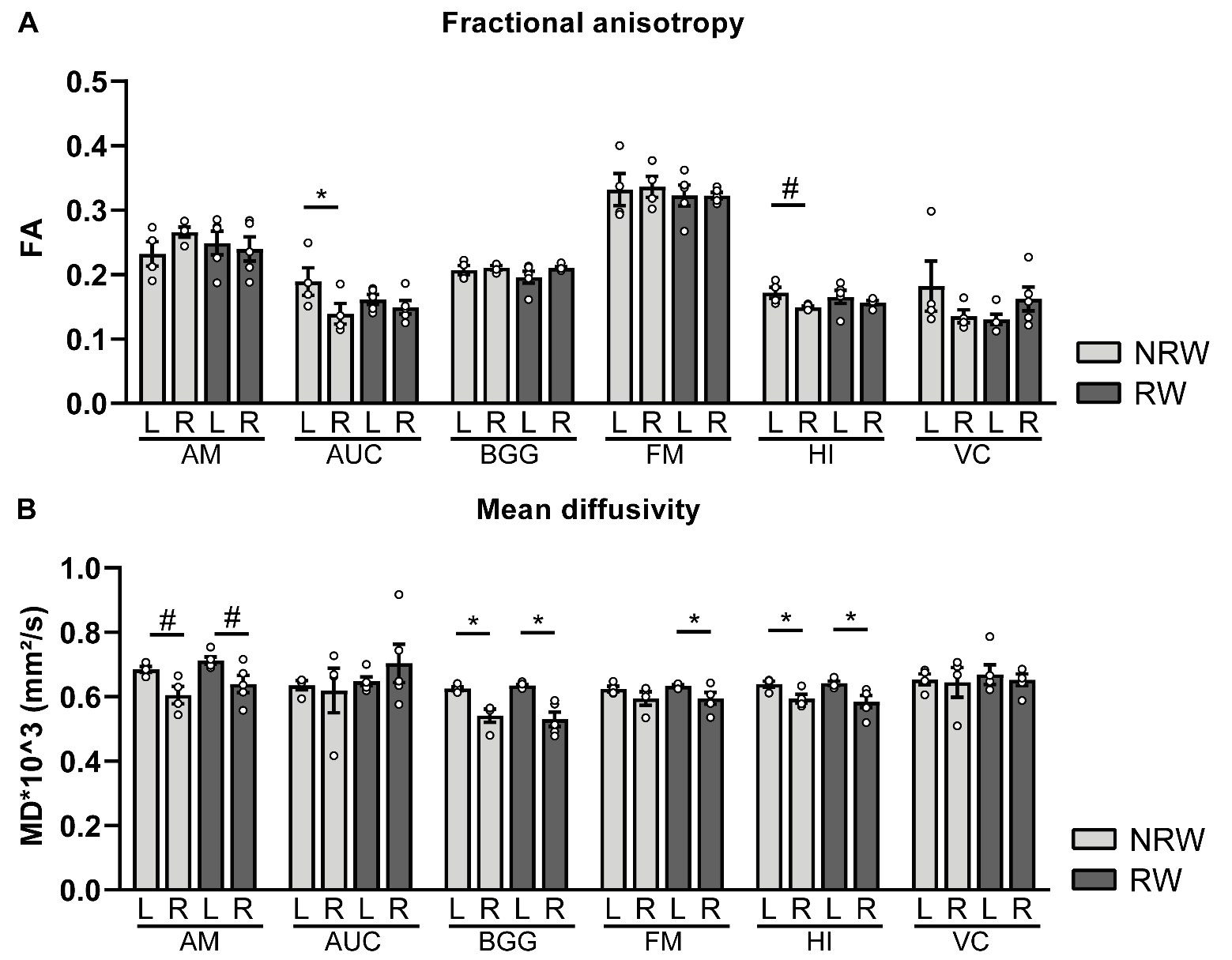


**Supplementary Figure 6** Diffusion tensor imaging measures (DTI) assessed at 6 days post stroke induction in the ipsilateral (R: right) and contralateral (L: left) hemisphere of running wheel (RW) and no running wheel (NRW) mice. Fractional anisotropy and mean diffusivity were assessed in different regions of interest (AM: Amygdala, AC: Anterior commissure, AUC: Auditory cortex, BGG: Basal ganglia, EC: External capsule, FI: Fimbria, FM: Forceps minor, HI: Hippocampus, IC: Internal capsule, MC: Motor cortex, OT: Optic tract, SSC: Somatosensory cortex, VC: Visual cortex). (A) Among NRW mice the ipsilateral auditory cortex (F(1,3)=17.6, p<0.025) and the ipsilateral hippocampus (F(1,3)=7.6, p<0.071) showed lower FA than the corresponding contralateral ROI. (B) MD values were decreased in the ipsilateral amygdala (trend: NRW: F(1,3)=9.3, p<0.055; RW: F(1,4)=5.9, p<0.072), basal ganglia (NRW: F(1,3)=24.7, p<0.016; RW: F(1,4)=25.5, p<0.007), and hippocampus (F(1,3)=11.9, p<0.041; RW: F(1,4)=9.1, p<0.039) when compared to the contralateral hemisphere of the corresponding regions. In the ipsilateral forceps minor RW showed higher lower MD values then in the contralateral hemisphere (F(1,4)=6.5, p<0.063). RW: N=5, NRW: N=4; values represented in mean ± SEM #, 0.05 < p < 0.08 (tendency); *, p ≤ 0.05.


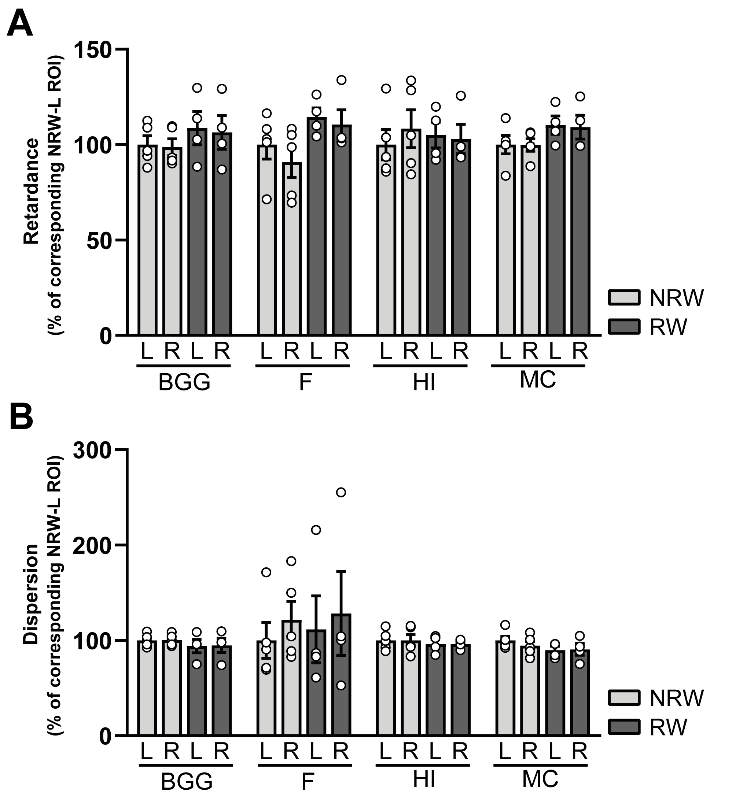


**Supplementary Figure 7** Relative dispersion and retardance values assessed with polarized light imaging (PLI) in several ROI of the ipsilateral (R: right) and contralateral (L: left) brain hemisphere (BGG: Basal ganglia, F: Fornix, HI: Hippocampus, MC: Motor cortex) of running wheel (RW) and no running wheel (NRW) mice. Both dispersion and retardance values were normalized relative to the respective contralateral ROI of the NRW mice. (A, B) No differences in retardance and dispersion were found in the shown areas. RW: N=5, NRW: N=4; values represented in mean ± SEM.


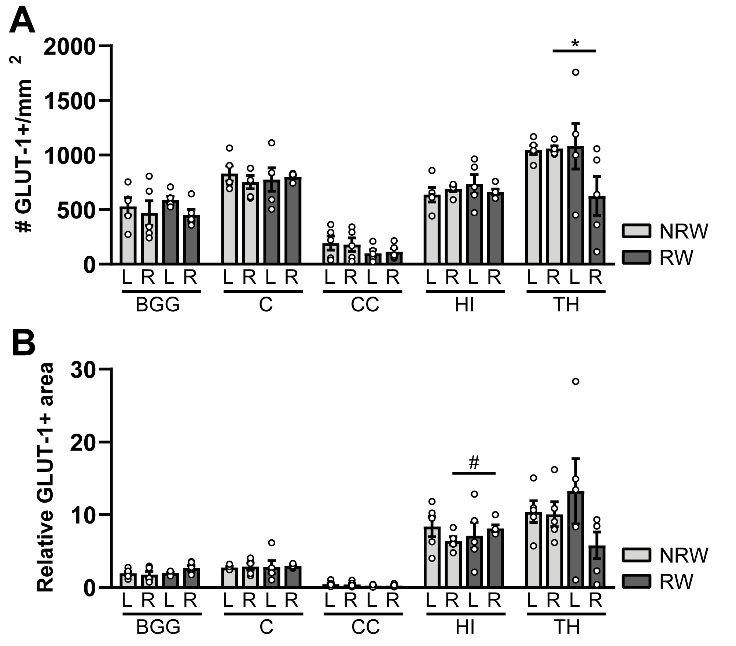


**Supplementary Figure 8** Immunohistochemical staining of GLUT-1 was performed to assess cerebrovascular integrity in the ipsilateral (R: right) and contralateral (L: left) hemisphere of running wheel (RW) and no running wheel (NRW) mice. (A) In the ipsilateral thalamus less GLUT-1+ blood vessels were measured in RW mice when compared to NRW mice (F(1,8)=5.9, p<0.042). Moreover, the ipsilateral basal ganglia of the RW exhibited less GLUT-1+ blood vessels than in the contralateral basal ganglia (F(1,4)=17.1, p<0.014). (B) The relative GLUT-1 positive area tended to be larger in the ipsilateral hemisphere of RW mice when compared to the same region in NRW animals (F(1,8)=5.3, p<0.051). RW: N=5, NRW: N=5; values represented in mean ± SEM #, 0.05 < p < 0.08 (tendency); *, p ≤ 0.05.

**Supplementary Table 4** Summary of all significant results in the paper. Abbreviations: running wheel (RW), no running wheel (NRW), diffusion tensor imaging (DTI), digital ventilated cages (DVC), fractional anisotropy (FA), mean diffusivity (MD), resting state functional MRI (rsfMRI), glucose transporter-1 (GLUT-1), ionized calcium-binding adapter molecule 1 (IBA-1), polarized light imaging (PLI).

| **Parameter** | | | **Results** |
| --- | --- | --- | --- |
| **Relative weight loss** | | | ↓in both groups D1 (at day 6 not significant anymore) |
| **Daily food intake** | | | ↑ in both groups at D6 |
| **DVC** | | **Activity** | ↑ in RW mice at D6 during nighttime; (↓) in both groups at D3 during nighttime |
|  |  | **Distance** | ↑ in RW mice at D5 during daytime |
|  |  | **Occupancy** | ↑ in RW at D6 during nighttime |
|  |  | **Laterality** | ↑ in RW at D1 during daytime; (↑) in RW at D5 during daytime; (↑) in RW at D1 during nighttime; ↑ in RW at D2 during nighttime; ↑ in RW at D5 during nighttime |
| **Open field** | **Automatic scoring** | **Distance** | = |
|  |  | **Velocity** | = |
|  | **Body angle state** | **Turns** | ↑ in the NRW mice turns to the left |
| **Infarct size** | | | = |
| **Cerebral blood flow** | | **Hippocampus** | ↓in both groups in the right hemisphere compared to the left hemisphere |
|  |  | **Thalamus** | = |
|  |  | **Motor cortex** | ↑ in RW mice than in NRW mice; ↓in NRW mice in the lefthemisphere compared to the left hemisphere |
|  |  | **Sensory cortex** | (↑) in RW mice than in NRW mice; ↓in both groups in the right hemisphere compared to the left hemisphere |
|  |  | **Basal Ganglia** | ↑ in RW mice than in NRW mice; ↓in both groups in the right hemisphere compared to the left hemisphere |
| **rsfMRI** | | **Partial** | ↑ in RW mice than in NRW mice: left auditory cortex and right somatosensory cortex |
| **DTI** | **FA** | **Motor cortex** | = |
|  |  | **Optic tract** | ↑ in RW mice than in NRW mice |
|  |  | **Somatosensory cortex** | = |
|  |  | **Anterior commissure** | = |
|  |  | **External capsule** | ↓in both groups in the right hemisphere compared to the left hemisphere |
|  |  | **Fimbria** | (↑) in NRW mice in the right hemisphere compared to the left hemisphere |
|  |  | **Internal capsule** | (↑) in RW mice in the right hemisphere compared to the right hemisphere in NRW; ↑ in NRW mice in the right hemisphere compared to the left hemisphere |
|  | **MD** | **Motor cortex** | ↓in RW mice in the right hemisphere compared to the left hemisphere |
|  |  | **Optic tract** | ↓in RW mice in the right hemisphere compared to the left hemisphere |
|  |  | **Somatosensory cortex** | = |
|  |  | **Anterior commissure** | = |
|  |  | **External capsule** | ↓in RW mice in the right hemisphere compared to the left hemisphere |
|  |  | **Fimbria** | ↑ in RW mice than in NRW mice |
|  |  | **Internal capsule** | ↓in both groups in the right hemisphere compared to the left hemisphere |
| **PLI** | **Dispersion** | **Anterior commissure** | = |
|  |  | **Cerebral Peduncle** | = |
|  |  | **Corpus callosum** | (↓) in RW mice in the right hemisphere compared to the right hemisphere in NRW |
|  |  | **External capsule** | (↑) in NRW mice and ↑ RW in the right hemisphere compared to the left hemisphere |
|  |  | **Fimbria** | ↓in RW mice than in NRW mice |
|  |  | **Internal capsule** | ↑ in NRW mice in the right hemisphere compared to the left hemisphere |
|  |  | **Optic tract** | (↓) in RW mice in the left hemisphere compared to the left hemisphere in NRW |
|  |  | **Sensory cortex** | ↑ in NRW mice in the right hemisphere compared to the left hemisphere |
|  |  | **Thalamus** | ↓ in RW mice in the left hemisphere compared to the left hemisphere in NRW |
|  | **Retardance** | **Anterior commissure** | ↑ in RW mice than in NRW mice |
|  |  | **Cerebral Peduncle** | ↑ in both groups in the right hemisphere compared to the left hemisphere |
|  |  | **Corpus callosum** | ↑ in RW mice than in NRW mice |
|  |  | **External capsule** | ↑ in RW mice than in NRW mice; ↓in NRW mice in the right hemisphere compared to the left hemisphere |
|  |  | **Fimbria** | = |
|  |  | **Internal capsule** | = |
|  |  | **Optic tract** | ↑ in RW mice than in NRW mice |
|  |  | **Sensory cortex** | = |
|  |  | **Thalamus** | ↑ in RW mice in the right hemisphere compared to the right hemisphere in NRW; ↑ in RW mice in the right hemisphere compared to the left hemisphere |
| **GLUT-1** | **Cortex** | **Mean grey value** | ↑ in NRW mice in the right hemisphere compared to the left hemisphere |
|  | **Hippocampus** |  | = |
|  | **Thalamus** |  | ↑ in RW mice than in NRW mice; (↑) in RW mice in the right hemisphere compared to the left hemisphere |
|  | **Corpus callosum** |  | = |
|  | **Basal ganglia** |  | ↑ in RW mice in the right hemisphere compared to the left hemisphere |
| **IBA-1** | **Hippocampus** | **#/mm^2^** | = |
|  |  | **Relative area** | ↑ in RW mice in the right hemisphere compared to the left hemisphere |
|  | **Thalamus** | **#/mm^2^** | ↑ in both groups in the right hemisphere compared to the left hemisphere |
|  |  | **Relative area** | (↑) in RW mice than in NRW mice; ↑ in RW mice in the right hemisphere compared to the left hemisphere |
|  | **Cortex** | **#/mm^2^** | ↑ in RW mice in the right hemisphere compared to the left hemisphere |
|  |  | **Relative area** | (↑) in RW mice in the right hemisphere compared to the left hemisphere |
|  | **Corpus callosum** | **#/mm^2^** | = |
|  |  | **Relative area** | = |
|  | **Basal ganglia** | **#/mm^2^** | ↑ in both groups in the right hemisphere compared to the left hemisphere |
|  |  | **Relative area** | ↑ in both groups in the right hemisphere compared to the left hemisphere |

**Materials and methods**

*Sample size calculation*

A final sample size of 5 mice per group was determined based on power calculation based on results from our previous study (Wiesmann et al., 2017). Here, we calculated functional connectivity averages and standard deviations of the Control (35.0g ± 1.4) and Fortasyn mice (37.6g ± 1.5) between right auditory and right somatosensory cortex being needed for the effect size calculation for this recent study (d=1.79). Although diet, and not exercise, was used as a treatment approach the experimental design is similar, since imaging parameters are the primary outcome measure in this study. A power analysis has been performed with alpha level (or the Type I error rate, α=0.05), statistical power (1-β, 0.80), an assumption of equal sized sample groups (N1 to N2 is 1), and the calculated effect size (1.79): n=5 per group (actual power: 0.93). We used n=8 Running wheel mice and n=7 No-Running wheel mice to ensure enough statistical power, since increased mortality was expected after stroke induction.

**References**

Wiesmann, M., Zinnhardt, B., Reinhardt, D., Eligehausen, S., Wachsmuth, L., Hermann, S., Dederen, P.J., Hellwich, M., Kuhlmann, M.T., and Broersen, L.M. (2017). A specific dietary intervention to restore brain structure and function after ischemic stroke. *Theranostics* 7**,** 493.
